# Metagenomic Analysis of Ready-to-Eat Foods on Retail Sale in the UK Identifies Diverse Genes Related to Antimicrobial Resistance

**DOI:** 10.3390/microorganisms13081766

**Published:** 2025-07-29

**Authors:** Edward Haynes, Roy Macarthur, Marc Kennedy, Chris Conyers, Hollie Pufal, Sam McGreig, John Walshaw

**Affiliations:** 1 Fera Science Ltd., York Biotech Campus, Sand Hutton, York YO41 1LZ, UK; roy.macarthur@fera.co.uk (R.M.); marc.kennedy@fera.co.uk (M.K.); chris.conyers@fera.co.uk (C.C.); hollie.pufal@newcastle.ac.uk (H.P.); sam.mcgreig@ukhsa.gov.uk (S.M.); john.walshaw@fera.co.uk (J.W.); 2 School of Natural and Environmental Sciences, Newcastle University, Newcastle upon Tyne NE1 4LB, UK

**Keywords:** high-throughput sequencing, next-generation sequencing, exposure modelling, antimicrobial resistance genes

## Abstract

Antimicrobial Resistance (AMR), i.e., the evolution of microbes to become resistant to chemicals used to control them, is a global public health concern that can make bacterial diseases untreatable. Inputs including antibiotics, metals, and biocides can create an environment in the agrifood chain that selects for AMR. Consumption of food represents a potential exposure route to AMR microbes and AMR genes (ARGs), which may be present in viable bacteria or on free DNA. Ready-to-eat (RTE) foods are of particular interest because they are eaten without further cooking, so AMR bacteria or ARGs that are present may be consumed intact. They also represent varied production systems (fresh produce, cooked meat, dairy, etc.). An evidence gap exists regarding the diversity and consumption of ARGs in RTE food, which this study begins to address. We sampled 1001 RTE products at retail sale in the UK, in proportion to their consumption by the UK population, using National Diet and Nutrition Survey data. Bacterial DNA content of sample extracts was assessed by 16S metabarcoding, and 256 samples were selected for metagenomic sequencing for identification of ARGs based on consumption and likely bacterial DNA content. A total of 477 unique ARGs were identified in the samples, including ARGs that may be involved in resistance to important antibiotics, such as colistin, fluoroquinolones, and carbapenems, although phenotypic AMR was not measured. Based on the incidence of ARGs in food types, ARGs are estimated to be present in a high proportion of average diets. ARGs were detected on almost all RTE food types tested (48 of 52), and some efflux pump genes are consumed in 97% of UK diets.

## 1. Introduction

Antimicrobial resistance (AMR) is increasingly recognised as a vitally important, global public health concern [1], potentially causing infectious diseases to become untreatable and making some medical procedures (e.g., anticancer therapy, organ transplant) unusable. This is especially important when considering the emergence of resistance to so-called critically important antimicrobials (CIAs) (e.g., [2]), which can be the last line of defence against bacteria already resistant to frontline antibiotics. The use of antimicrobials in the agrifood chain is known to lead to the evolution of AMR genes (ARGs), which may be transmitted to human pathogens or the human commensal microbiota [3,4].

An evidence gap exists about the extent to which consumption of foodstuffs contributes to antimicrobial resistance in the human microbiome, especially for ready-to-eat (RTE) products. These products are of particular interest because they are consumed without further cooking in the home. Without a cooking stage to damage or denature them, any AMR bacteria (or intact ARGs) present could contribute to the ARG content of the microbiome of the consumer.

RTE products also span a range of production techniques, which may differ in the extent to which they promote the evolution of AMR, based on differing antimicrobial inputs during production. Cooked, RTE meats and dairy products are both animal-based foods, and the animals involved may have been treated with antibiotics during the primary production process. Non-RTE meats including red meat [5] and poultry [6] are known to harbour AMR bacteria, and it is not currently known how prevalent AMR bacteria are on RTE meats on retail sale in the UK. Dairy animals can be exposed to antibiotics to treat diseases such as mastitis either therapeutically or prophylactically (e.g., via dry cow therapy [7]), and this may lead to the evolution of AMR. Fresh produce, including RTE fruits and vegetables, may also have been directly treated with antibiotics [8], indirectly exposed to antibiotics via soil amendments such as manure [9], or subject to co-selection for AMR from agrichemicals such as metals [10], which can lead to increased prevalence of AMR bacteria or ARGs on crops. Other RTE food types, such as seafood, also have considerable antimicrobial inputs during production, for example, in aquaculture [11]. Beyond primary production, slaughter/harvest and secondary processing of foods may contain sources of antimicrobial compounds, which may drive AMR evolution. For example, there is some evidence that biocides may lead to co-evolution of resistance to antibiotics [10,12], although the picture is still mixed [13,14]. Despite the varied routes by which ARGs may be introduced and selected for, there remains an evidence gap about the extent of ARG presence in RTE foods on retail sale.

Metagenomic sequencing involves non-targeted sequencing of DNA from a sample, and it can be used to identify ARGs that are present. This has several advantages over more traditional phenotypic screening for AMR bacteria. For example, ARGs can be detected regardless of the bacterium they are present in (including pathogenic and commensal species), and the DNA sequences generated can be screened against all ARGs present in the relevant database(s). Metagenomics is not in routine use for screening foods for ARGs [15] and has very rarely been used in RTE foods in any context (except, e.g., by Li et al. [16]). However, metagenomic data does not provide information about the expression of the ARGs detected or the presence of any resulting AMR phenotype, and as such, we confine our investigation to the identification of genes which may be involved in AMR rather than performing a microbiological risk assessment of phenotypic AMR or foodborne disease.

To begin to address the evidence gap around intake of ARGs from food, and to better understand the advantages and limitations of a metagenomic approach to ARG detection in RTE foods, we sampled products from a variety of RTE food categories (cooked meats; dairy products; fresh produce), weighted by UK consumption data. A subset of samples was selected for metagenomic sequencing to identify the presence of AMR genes, emphasising food types that were highly consumed and that had higher proportions of bacterial DNA, and concentrating on genes related to bacterial resistance to antibiotics. This data was combined with food consumption data from the National Diet and Nutrition Survey (NDNS) [17] to estimate the incidence of ARGs in UK diets. This provided important insights into the incidence of ARGs in RTE food on retail sale in the UK and about the limitations of metagenomic detection of ARGs.

## 2. Materials and Methods

### 2.1. Sampling Strategy

The sampling strategy was designed to sample the most consumed food types such that at least 90% of consumption of in-scope RTE foods was covered. Numbers of samples of each food type were in proportion to the amount of the food type in the average diet but with a minimum of five samples per food type. This resulted in 52 different food types being sampled, comprising 33 types of produce, 17 types of dairy, and 2 types of cooked meat. While fewer dairy types were sampled than produce types, more than half of the samples taken were dairy samples. Finally, the number of samples of each food type taken from each UK region was assigned in proportion to the region’s population. Ninety-five sets of duplicate samples were taken: two samples of the same product, from the same lot, were bought from the same location at the same time; samples were selected at random (by product type, region, and retail outlet) to be duplicated.

### 2.2. Sampling

Samples were collected by HallMark Veterinary & Compliance Services based on the sampling strategy and from the eight largest supermarket retailers. Samples were taken according to market share. For the purposes of sample handling, samples were divided into two broad categories—dairy and produce. Dairy samples (538 in number) were collected during the first half of the collection period, which ran from 17 June 2019 to 27 August 2019. Produce samples (504 in number) were collected in the second half of the collection period, which ran from 2 September 2019 to 28 October 2019. Upon receipt at the laboratory, all samples were handled while wearing disposable nitrile gloves.

### 2.3. DNA Extraction

#### 2.3.1. Dairy Samples

Samples were extracted on the day of receipt where possible. When this was not possible due to a large number of samples arriving, half of the samples were frozen for processing on the next day on which samples were not due to arrive.

For dairy samples, 2 × 1.8 mL aliquots were taken from each sample, 1 of which was frozen at −40 °C as a reserve sample. The samples were extracted using a Qiagen DNeasy^®^ PowerFood^®^ Microbial Kit (Qiagen Ltd., Venlo, The Netherlands) according to the Milk Extraction Protocol with the following modifications: initial centrifugation of the samples at 13,000× *g* was increased from 3 to 5 min minutes; after addition of 450 µL of Solution MBL (from the Qiagen kit), samples were incubated at 75 °C (instead of 65 °C) in a thermomixer for 5 min at 550 rpm; physical lysis in the PowerBead tubes (Qiagen Ltd.) was performed for 15 min (instead of 10 min). For each batch of samples processed, an extraction blank was included, which, in the case of the dairy samples, was 450 µL of Solution MBL added to a clean tube with no bacterial pellet and processed in the same manner as the dairy samples.

#### 2.3.2. Produce Samples

All produce samples, apart from cheese (included in the produce samples collection set for logistical reasons), were processed on the day of receipt. Cheese samples were stored at −40 °C as they required a different extraction protocol.

Samples were processed according to their sample type—see Table 1 below. Briefly, samples that were usually consumed whole were rinsed, and DNA was extracted from the rinsate. This was carried out in order to preferentially sample external bacteria and reduce contamination with host/matrix DNA. Samples that were usually consumed peeled were peeled, and the consumed flesh was then rinsed.

Solid samples were placed into ziplock bags, and 25 mL/50 mL of rinse buffer (1× TE with 1.2% (*v*/*v*) Triton X-100 and 3% (*v*/*v*) antifoam) was added. The bag was shaken gently (soft fruits/meats), or the fruit was rubbed in the buffer for approximately 30 s. The buffer was transferred to a 50 mL Falcon tube and centrifuged at 10,000× *g* for 10 min to obtain a pellet.

Liquid samples were centrifuged directly to obtain a pellet. For orange juice, which tended to generate more sediment than apple juice, 1 ml was centrifuged at 10,000× *g* for 10 min. For apple juice, 15 mL was centrifuged at 3430× *g* for 10 min (lower speed due to larger volume requiring a centrifuge and rotor with lower maximum speed).

All samples from this point underwent the same extraction procedure for Gram-positive bacteria using a Qiagen DNeasy Blood and Tissue Kit (Qiagen Ltd.) following the manufacturer’s protocol. For each batch of samples processed, an extraction blank was included, which comprised 1 mL of rinse buffer processed in the same manner as orange juice.

#### 2.3.3. Cheese Samples

The cheese samples were stored at −40 °C to enable them to be processed as one batch of 24 samples. The samples were thawed thoroughly, and 1 g was weighed into a 15 mL Falcon tube. Initial processing for DNA extraction occurred subsequently [18]. An extraction blank comprising 5 mL of 2% *w*/*v* sodium citrate solution was processed in the same manner as the cheese samples.

### 2.4. 16S Sequencing

#### 2.4.1. PCR Amplification

Prior to PCR, samples were distributed across 6 × 96-well plates for dairy samples and 6 × 96-well plates for produce samples, ensuring at least two samples of the same type would be on the same MiSeq run, and also that samples from a single type were distributed across at least two MiSeq runs, to account for any inter-run variation.

PCR was performed using 16Sv4 primers in order to amplify bacterial DNA. PCR reactions comprised 0.3 mM dNTPs, 0.3µM each of forward and reverse primer, and 0.6 units Phusion^®^ High Fidelity DNA Polymerase (New England BioLabs, Ipswich, MA, USA) in 1 × HF buffer and 1 µL of DNA extract as template in a total volume of 25 µL. A positive control sample of NGSgBlock (synthetic oligonucleotide encompassing primer binding sites for 16S and ITS primers) at 0.005 ng/µL and a PCR negative control comprising 1 µL of molecular-biology-grade water were also amplified alongside the samples for quality control purposes. Primers for 16Sv4 [19,20,21,22] were as follows:

Nex_16S_515F: (**TCGTCGGCAGCGTCAGATGTGTATAAGAGACAG**GTGYCAGCMGCCGCGGTAA);

Nex_16S_8067R: (**GTCTCGTGGGCTCGGAGATGTGTATAAGAGACAG**GGACTACNVGGGTWTCTAAT).

Nextera tag sequences are highlighted in bold. These allow the index tags to be added to the samples during library preparation to allow for discrimination of individual samples following sequencing.

Samples were amplified with the following ‘touch down’ thermocycling conditions on a BioRad C1000 thermal cycler:

Initial denaturation at 98 °C for 2 min, followed by 22 cycles of denaturation at 98 °C for 20 s, primer annealing at 65 °C for 45 s decreasing 0.5 °C per cycle down to 54 °C, extension at 72 °C for 60 s, then a further 8 cycles of 98 °C for 20 s, 54 °C for 45 s, 72 °C for 60 s, followed by a final extension at 72 °C for 10 min and hold at 4 °C. The total number of cycles was 30.

Following thermocycling, amplification success was measured by visualisation of amplicons on agarose gels containing 0.1 µg/mL ethidium bromide (Sigma, Merck Life Science UK Ltd., The Old Brickyard, New Road, Gillingham, Dorset, UK).

The proportion of samples with a visible band after PCR amplification was relatively low (55% of dairy samples generated visible amplicons, 65% of produce samples generated visible amplicons). However, with no a priori information about what proportion of samples should generate high-quality amplicons, all samples were taken forward for sequencing.

#### 2.4.2. Sequence Library Preparation

Library preparation took place based on the Illumina protocol for 16S Metagenomic Sequencing Library Preparation. Firstly, the remaining 20 µL amplicon for each sample underwent a size-selection magnetic bead clean-up to remove unincorporated PCR components and any small non-specific products (e.g., primer-dimers). Briefly, 16 µL AMPure XP (Agencourt) magnetic beads (Beckman Coulter Inc., Diagnostics Division Headquarters, 250 South Kraemer Boulevard, Brea, CA, USA) were added to 25 µL PCR reaction and processed as per the manufacturer’s instructions.

Index PCR was performed using Illumina Nextera XT Index Kit v2 dual index adapters (Illumina Inc., 5200 Illumina Way, San Diego, CA, USA). PCR reactions comprised 0.3 mM dNTPs, 5 µL each of N7 and S5 adaptors from the index kit, 1 mM MgCl_2_, and 1 unit Phusion^®^ High Fidelity DNA Polymerase (New England BioLabs) in 1 × HF buffer and 5 µL of cleaned amplicon as a template in a total volume of 50 µL. An index negative was included, which comprised 5 µL of molecular-biology-grade water. Samples were index amplified with the following thermocycling conditions on a BioRad C1000 thermal cycler (Bio-Rad Laboratories Inc., 1000 Alfred Nobel Drive, Hercules, CA, USA).

Initial denaturation at 95 °C for 3 min, followed by 8 cycles of denaturation at 95 °C for 30 s, adapter annealing at 55 °C for 30 s, and extension at 72 °C for 30 s, followed by a final extension at 72 °C for 5 min and hold at 12 °C.

Indexed samples (or ‘libraries’) then underwent a second magnetic bead clean to remove unincorporated PCR components. The protocol was as for the first-round bead clean with the exception of the bead volume and elution volume. For the index PCR bead clean, 56 µL AMPure XP magnetic beads were added to the 50 µL index PCR reaction, and following bead drying, 27.5 µL of molecular-biology-grade water was added to resuspend the beads with a final volume of 25 µL of supernatant being transferred to a clean 96-well plate following bead pelleting.

The qualities of the libraries were then assessed by quantifying all libraries using either a Qubit™ dsDNA HS Assay (Invitrogen, 5781 Van Allen Way, Carlsbad, CA, USA) and measuring the library concentration on a Qubit™ fluorometer or a Quant-iT™ Picogreen™ dsDNA Assay Kit (Invitrogen) and measuring the library concentration on a Fluoroskan Ascent plate reader (Thermo FisherScientific, Wyman Street, Waltham, MA, USA). In addition, a selection of high- and low-quantifying libraries plus all controls (i.e., PCR positive, PCR negative, extraction blanks, index PCR negative) were run on an Agilent Technologies TapeStation 2200 (Agilent Technologies, 5301 Stevens Creek Blvd, Santa Clara, CA, USA) using HS D1000 tapes, size ladder, and sample buffer.

#### 2.4.3. MiSeq 16S Amplicon Sequencing

Libraries were sequenced on an Illumina MiSeq sequencer (Illumina, Inc.) using a MiSeq Reagent Kit V3. A total of 10 pmol of sample pool and 10% PhiX was loaded on to the machine for sequencing.

### 2.5. Sample Selection for Metagenomic Sequencing

Based on the results of the 16S metabarcoding analysis, food types were separated into food types with a high proportion of reads of bacterial origin, food types with a low proportion of reads of bacterial origin, and food types showing a continuum of bacterial–host DNA. The level of bacteria was defined by inspecting the distribution of bacterial and host reads across each sample type (see Section 3.3). ‘Low bacteria’ was assigned to food categories that had the majority of the samples with 75% or greater host reads, ‘High bacteria’ with those with the majority of samples with 75% of greater bacterial reads, and a continuum when bacterial reads were distributed across a wider range of percentages. Food types for metagenomic sequencing were then weighted such that 80% of samples came from the high-bacteria and continuum food types, and 20% came from the low-bacteria food types. A total of 256 samples were randomly allocated from within these types. This was carried out in order to focus on those foods which were most likely to give information about ARG content (note, NOT those which were most likely to have ARGs), and not on those foods where we were unlikely to obtain information on the ARG content (note, NOT those which were unlikely to have ARGs). This is somewhat analogous to limits of detection of tests—focusing only on situations where our test had a better limit of detection.

This design still permitted the sequencing of a minority of samples from the foods where we were unlikely to obtain much information about the ARG content (i.e., those with low proportions of bacterial 16S sequences), but it was felt that (i) it would be useful to some extent for checking the validity of our upstream decision making and (ii) that this would allow some highly consumed food types to be sequenced that would otherwise be omitted (e.g., bananas).

Where available, a duplicate sample pair was sequenced for each food type. We also sequenced ten samples that were technical fails for 16S sequencing to check whether failure of 16S sequencing is a good predictor of failure of metagenomic sequencing.

### 2.6. Metagenomic Sequence Library Preparation and Sequencing

The 256 samples selected for NovaSeq sequencing underwent Illumina Nextera Flex library preparation (now Illumina DNA Prep) following the Illumina protocol (document 1000000025416 v 7 May 2019). Briefly, the DNA underwent fragmentation and addition of Nextera tags in a single enzymatic step (the Nextera tags being the same sequence as above on the 16S primers). Unique dual-index adaptors were added via a PCR reaction, in a similar way to the amplicon index PCR, followed by a double-sided bead purification of the libraries to remove any very small or very large fragments. The libraries were quantified as before using a Quant-iT™ Picogreen™ dsDNA Assay Kit (Invitrogen) and measuring the library concentration on a Fluoroskan Ascent plate reader (Thermo Scientific). In addition, a selection of high- and low-quantifying libraries plus the index PCR negative were analysed on the Agilent TapeStation using HS D5000 tapes (Agilent Technologies), size ladder, and sample buffer.

Once the quality of the libraries had been assessed, the libraries were pooled in equimolar amounts to create a 20 nM library pool in a 1 ml total volume. The pool was quantified using a Qubit™ dsDNA HS Assay (Invitrogen) to determine the actual concentration, and the average size of the pool was determined by running the pool on the TapeStation (Agilent Technologies).

Following confirmation of the quality and concentration of the library, the prepared sequence library was couriered on ice to Newcastle University. Clustering QC was carried out on an Illumina MiSeq using Reagent Kit V2 Nano (Illumina Inc.). The library was then prepared for sequencing according to the NovaSeq 6000 Sequencing System Guide using two NovaSeq S2 300 cycle (2 × 150 bp) Flowcells (Illumina Inc.).

### 2.7. Bioinformatic Analysis

#### 2.7.1. 16S Metabarcoding

A total of 1001 paired-end fastq files were generated over 6 Illumina MiSeq runs and subsequently imported into the Qiime2 [23] software for analysis. Sequences were trimmed by the cutadapt software [24], which identifies primers in the sequences and trims them. In addition to this, any sequences which were shorter than 50 nucleotides were removed so as to exclude erroneous sequences from further analysis. Sequences were then denoised using Dada2 [25], which attempts to correct sequencing errors, where possible, to determine real biological sequence variants (Amplicon Sequence Variants, ASVs). This process also joins and quality-filters the sequences, in addition to checking for chimeras in the ASVs. Any sequences which were shorter than 160 nucleotides were discarded. This threshold was chosen so that host sequences (such as cow or pig mitochondria) would be included in the final dataset, which can be used to infer the relative proportion of bacteria to host DNA. Chimeras were then checked for again using the Vsearch tool [26], and a feature table and a set of representative sequences were produced. Any samples with fewer than 3000 reads were classified as ‘fails’ and removed from further analysis. Features were then classified using Vsearch, with the Silva database (version 138) providing taxonomic information. The relative abundance matrix produced was used to identify food categories by grouping the assignments into bacteria and host categories, where host was defined as either chloroplast or eukaryote taxonomic assignments.

#### 2.7.2. Quality Control and Host Sequence Removal from Metagenomics Data

A total of 256 paired-end fastq files were generated on each of two Illumina NovaSeq S2 300 cycle (2 × 150 bp) Flowcells. Each sample was trimmed to a quality score of 20 and a minimum length of 50 using sickle [27], and then reads originating from the same sample from each flow cells through out were combined. In order to remove sequencing reads which originated from a non-bacterial source, i.e., the food “host genome”, the trimmed sequence data was then compared to a relevant host genome, where available, using the bwa [28] software, which performs “mapping” of reads to reference sequences. Samples which had no suitable host genome readily available were mapped to a related genome instead (see Supplementary Information S4 for genomes used). Unmapped reads were then extracted using the samtools [29] software.

#### 2.7.3. Identification of ARG Sequence Fragments in the Metagenomics Data

Given that a generally high frequency of undetected ARGs (false negatives) might be expected, especially in samples yielding relatively few bacterial reads, the most severe criteria for identifying reads of ARG origin would not have been appropriate. Nonetheless, since the modelling methods (Section 2.8. Exposure Modelling) used ARG presence as the input, rather than relative frequencies, it was important to ensure that most predictions were of high confidence. We therefore implemented an approach which applied appropriate constraints. As the basis of the ARG predictions, we used the RGI software and its associated database, the CARD [30], but we then applied a number of detailed filters to these results.

#### 2.7.4. RGI/CARD Analysis

We downloaded RGI (version 5.1.0) and CARD (3.0.8) and associated Resistomes, Variants, and Prevalence data (“WildCARD”, 3.0.6) on 11 May 2020 [30].

The CARD data itself is intensively curated, consisting of around 2600 sequences (v. 3.0.8) whose AMR nature is well-characterised. Given the additional strict filters which we subsequently applied to the RGI results, in combination with the anticipated breadth of food types and sequences that might be encountered in such a large number of samples, it was important to include the WildCARD reference data (around 150,000 sequences). These are themselves the result of the curators previously applying the RGI/CARD software to public sequence data, enforcing strict criteria.

RGI 5.1.0 offers a choice of mapper; we selected the BowTie2 [31] option. RGI limits the mapping of each read pair to only a single (or no) location (in one of the reference gene sequences) in the alignment file. Accordingly, each sample’s QC-filtered, host-DNA-filtered paired reads were analysed using an rgi bwt command using the options—aligner bowtie2—including wildcard. Full details are in Supplementary Information S6.

#### 2.7.5. Filtering of RGI Results

For our subsequent analyses, other than to obtain some basic statistics of unique ARG names and protein sequence identities, we did not use the principal final outputs of rgi bwt; these are two tables for each sample, whose rows represent ARG names or particular variants (alleles). Each column contains various metrics describing the quality of the matches between the reads and the ARGs. Therefore, these metrics are averages over all the reads in the sample matched to that ARG or are represented as a range, such as the Reference Allele(s) Identity to CARD Reference Protein (%) column (e.g., ‘60.82–82.06’).

Instead, we applied a series of filters as follows:

- The “standard” filters consisted of an analysis of every mapped read in order to identify the least convincing matches and discard them.
- For one particular category of ARGs only, we subsequently applied a nucleotide sequence identity filter (the “variant/mutant type” filter).
- Finally, for each ARG, we applied a filter defined by the number of read pairs matched to it within each sample.

Standard filters: In more detail, the standard filters assessed several properties of each alignment of read to reference. With one exception, the metrics were calculated independently for the forward (R1) and reverse (R2) reads of each pair. The read-to-reference mappings’ properties can be broadly categorised as follows:

- Location of matching and mismatching read segments;
- Lengths of matching read segments and any mismatching segments;
- Uniqueness of the mapping;
- Plausibility of the sequences themselves irrespective of whether the read and reference sequences were very similar;
- Sequence identity of the matching read segments.

Locations of segments: Each ARG-mapped read can be considered to consist of one or more of three types of segments: matched, unmatched, and overhanging. The matched and unmatched segments collectively constitute that part of the read which notionally is expected to have been aligned to the reference, whereas the overhanging segments would not be expected to be, as follows. In the output mapping (SAM/BAM) files, commonly segments of the reads (at either or both ends) were clipped off and omitted from the alignment by the mapping software, as they do not sufficiently match the reference sequence. This can be due to “overhangs”, where the clipped segment represents genomic sequence beyond one end of the gene. A different reason for clipping is that the segment is locally too dissimilar to the reference; thus, the missing segment is “unmatched”. The segment included in the alignment (which may be the entire read) is “matched”. Matched segments are not necessarily identical to the aligned part of the ARG, nor should they be expected to be in every case. Unless unmatched regions are short, they indicate that the read overall does not represent a good enough match with the reference ARG, irrespective of how well the matched segment aligns.

Lengths of segments: Matched, overhanging, and unmatched segments are illustrated in Supplementary Information S5. Irrespective of the presence of unmatched or overhanging segments, the matched segment of each read needs to be of sufficient length to have discriminatory value, in terms of uniquely identifying an ARG. We also considered the relative length of the total matched read segment(s) in comparison with the total mapped + unmapped length. Excluding any overhanging segment(s) from the calculation, the matched segment was required to constitute at least 75% of the total length. The absolute length of the matched segment was required to be a minimum of 45 bp. Moreover, the total length of the two matched segments of each pair of reads was required to be a minimum of 75 bp, irrespective of whether one or both reads had been mapped at all. That is, when only one read had been mapped, the match would be at least 75 bp, thus coding for a minimum of 25 amino acids (assuming it is a coding sequence). When both reads had been mapped, the matched segments would necessarily sum to at least 90 bp, i.e., a minimum coding of 30 amino acids. This summed match–segment length was the sole criterion which involved the reads treated as a pair, with all others applied independently to each read.

Uniqueness: Some reads may equally well match multiple reference sequences; whether or not a read was uniquely maximally mappable (there is a single reference location with the maximum alignment score) is apparent in the MAPQ score assigned by BowTie2. Irrespective, RGI ensures that only a single mapping (or no mappings) is present for each read, indicating that for some reads, other mapped locations might have been equally valid. We discarded these ambiguous reads based on MAPQ scores of 0 or 1, even though this may lead to some false negatives.

Plausibility of read and matching reference sequences: Potentially, false positives may result from lengthy homopolymeric sequence segments and also long segments of very low complexity (e.g., dominated by one nucleotide base but interspersed with occasional instances of a different base). In terms of absolute frequencies, such segments are not rare in the sequence datasets and are a known issue (especially with poly-G) with two-colour sequencing platforms including NovaSeq [32].

Even if the reference database contained only a single long homopolymer sequence, assumed erroneous, then in a large high-throughput sequencing dataset, instances of the same homopolymer sequence are likely, which will match the reference well. This will result in an inflated count of this reference and, indeed, often a false positive in terms of presence versus absence. Low-complexity segments can also make very good matches with homopolymers (or each other) even if not completely identical. We therefore discarded any matches where the matched read segment contained homopolymers of longer than 12 bp.

Whereas homopolymers in mapped read segments are straightforward to detect, low-complexity sequences require a different approach such as a sequence entropy calculation (here, we used an entropy calculation on the overall proportion *p* of each base in the matched read segment, i.e., ∑ − *p_x_* ln(*p_x_*) for *x* = { A, C, G, T }; we discarded matches with entropy < 1.1).

Sequence identity of the matching read segments: In the general case, we did not impose any minimum sequence identity for the matches of reads to reference ARGs, largely because the other filters described are sufficiently stringent such that those reads which pass can be expected to usually have high identity with their reference ARG. Crucial exceptions are those gene variants whose resistance phenotypes depend on the presence of a few (possibly even one) point mutations compared to other versions of the gene (which are not AMR genes). This type of ARG is annotated in CARD with the Antibiotic Resistance Ontology (ARO [30]) “antibiotic resistant gene variant or mutant” ARO:0000031 (or by one of its descendant terms in the hierarchical ontology, which we accordingly parsed), and we required exact matches for these.

Many ARG types are subject to the usual considerations for inferring gene/protein function generally. Usually, thresholds of a relatively low sequence identity are sufficient for identifying genes of a particular function. Normally, the same function would be indicated by around 40% protein sequence identity or higher, with broader functional classes inferable from somewhat lower identity levels [33]. Gene sequences are generally less conserved (thus, a lower sequence identity) than the proteins they encode due to the redundancy of the genetic code. However, the same function does not necessarily equate to the “same” gene, and equivalent genes in closely related species (essentially, orthologues) will usually have high identity. Conversely, same-function, same-named genes/proteins in more distant relatives may have low identities, in some cases < 40%. A further overarching consideration is that the notional same-gene or same-function identity levels discussed apply to full-length sequences; all else being equal, higher identity levels would be ideal for inferences made from short fragment matches, such as metagenomics reads. A counterpoint is that within an alignment of full-length genes of a given identity, shorter segments may have considerably lower identity. For example, the coding sequences of *acrB* of *Escherichia coli* and *Salmonella enterica* code for proteins of 95% identity, with nucleotide identity of around 86%, but some 150 bp segments within are only 77% identical (e.g., GenBank accessions M94248.1 and MH933962.1). Yet, such considerations are further confounded in other ARG types by some instances of homologues of high sequence similarity which do not confer a full drug-resistance function [34,35]. These cases are not limited to the “gene variant or mutant” types.

Overall, there are thus no strict rules, and any identity thresholds imposed could be considered rather arbitrary; both tightening and loosening the stringency have disadvantages and advantages.

Independent calculation of sequence identity: To provide context to the results, we nonetheless performed independent pairwise local alignments (using the Smith–Waterman algorithm, via the water program of the EMBOSS package [36]) of all filter-passing reads with their reference ARGs to provide an identity distribution. The statistic we calculated was the percentage of the original total length of the read which was aligned to an identical nucleotide in the reference sequence. In effect, this is a worst-case scenario and differs from the percentage identity of the local alignment, which is calculated only from the segments of the read and reference which are included in the alignment (poor-matching segments of the read may be excluded completely).

In contrast to the general case, we used the calculated identities to filter those ARGs of the “resistant gene variant or mutant” category. By insisting on 100% sequence identity between the read segments and the references with which they matched, we avoided one kind of false positive, involving a high-similarity match which is nonetheless non-identical and lacks the resistance mutations. However, this is inherently recognisable only where the gene segment sequenced in the short-read pair encompasses at least one of the mutations. If the reads’ sequences span only a region that is identical between the susceptible and resistant forms, then a false positive can still result. Conversely, discarding such matched regions (by ignoring all variant/mutant types) would risk false negatives for these resistant variants.

We also determined the number of total named ARGs identified which belonged to the ARO:0000031 class and the samples in which they were positive with, and without, the 100% identity requirement (with all other filters applied in both cases).

In summary, we used the following quality criteria to determine retention in the filtering procedure. Unless otherwise stated, these criteria apply to the matched (mapped) segments of the reads (not necessarily the whole read).

- Absence of any homopolymers longer than a maximum threshold (12 bp);
- Sequence entropy of a magnitude greater than a minimum threshold (1.1);
- MAPQ score of at least a minimum threshold (2);
- Total length of all unmatched segments does not exceed a threshold proportion (25%) of the total unmatched + matched segments’ length;
- Matched segment length of at least a minimum threshold (45 bp);
- Total matched segment length of both reads of the pair of at least a minimum threshold (75 bp);
- Conditional upon the AMR type (as annotated by the ARO term), a minimum percentage identity between the matched segment and the aligned reference segment (if the ARG is annotated as ARO:0000031 or as any descendant ARO term, then the minimum identity is 100%; for all other ARGs, no minimum identity is required).

Observation frequency filter: We required each ARG to have been identified by at least two independent matches (read pairs) per sample (subsequent to all of the previous filters), so those represented by only a single read pair were removed from that sample’s results. It is possible for only one read of a pair to match an ARG (if it is at or near one end of the reference sequence; the paired read may match nothing, or even a different ARG). An ARG is treated as positive for a read pair if either or both of the reads match it, subject to the filtering criteria.

Availability of code: The filtering methods were implemented in analysis scripts along with pipeline scripts, available at https://gitlab.fera.co.uk/jwalshaw/argisamfilter, accessed on 22 May 2025. Refer to Supplementary Information S1 for further details.

### 2.8. Exposure Modelling

#### 2.8.1. Dietary Consumption Data

Data from the UK NDNS were extracted from the UK Data Archive [17]. The data included the same year 1–8 survey records as used in prioritising the sampling plan, collected between 2008/09 and 2015/16, plus the more recently added year 9 records (2016/17). Sample weightings were recalculated to account for the differences in sample sizes between years. The combined dietary dataset contains 13,350 individuals that are representative of the UK population. The consumptions of RTE items were extracted for each individual to provide information about the combinations of those items consumed per person.

#### 2.8.2. Assumptions Underlying the Sampling Design

The sampling strategy is described above. The total number of samples is 256. While fewer types of dairy were sampled than types of produce, more than half of the samples taken were dairy samples. The number of samples of each food type taken from each UK region was in proportion to its population. Samples were taken from the eight largest supermarket retailers according to market share. Hence the collection of samples was designed to be and is assumed to be representative of the overall UK population consumptions of in-scope RTE products in the dairy, produce, and meat categories.

#### 2.8.3. Possible Measures Related to AMR Intakes in the UK Diet

We can refer to intake in a single RTE food type as the incidence and prevalence of AMR genes in that food. In order to compare with other food types, the measures used should be consistent between foods.

For incidence, we identify the ARGs found at least once within the samples of a single food type (incidence per food) or found at least once across samples of all types (incidence in UK diet).

For prevalence, there are two possible quantities of interest. The first is the proportion of retail samples of individual ready to eat food types (e.g., what proportion of semi-skimmed milk samples in the UK contain a particular ARG or ARG type). The second is the prevalence in the UK diet overall (what proportion of individuals are exposed to a particular ARG or ARG type). Prevalence in the ready-to-eat portion of the UK diet is estimated from multiple foods and accounts for the consumption amounts of each food type as recorded in the UK dietary survey (NDNS). The two measures of intake defined here can be further refined by considering subsets of ARGs according to classifications of interest. Both individual ARGs and ARG classes were analysed with separate intake calculations.

#### 2.8.4. Incidence Summaries at Sample/Food Level

First, we estimate the proportion of samples (of a given food type) containing a specific ARG. This is consistent with the previous literature on measurement of AMR in foods. After calculating the incidence of individual ARGs in all samples (and within a food type), the following summaries can be derived by simple aggregation

- Number of ARGs in each single sample and the between-sample range seen in this number.
- Number of unique ARGs or ARG families found across all samples of a given food type.

#### 2.8.5. Incidence: Total UK Diet

The 52 sampled food types are consumed within the UK and appear in the NDNS consumption diary data. Therefore, the incidence and the number of distinct ARGs in the diet can be estimated directly from the data. A lower-bound estimate is simply the number of unique ARGs combined across all the collected samples. It is possible that others are present in the wider diet but not measured due to the limited sample sizes.

#### 2.8.6. Prevalence Calculations, per Food

For a single food, we can compute the proportion of samples containing individual ARGs (direct count of samples in which the ARG appears, relative to the total number of samples obtained for the food type). This is important for understanding which RTE food types in UK retail are potential sources of AMR and the relative levels of different AMR genes.

#### 2.8.7. Frequency (Relative Number of Samples) of a Given ARG in a Food-Specific Dataset

These can be compared across food categories by plotting or tabulating the data (e.g., see Section 3.5).

#### 2.8.8. Prevalence Calculations, Population Level

When considering a particular ARG, the prevalence is defined as the proportion of UK individuals whose diet contains that ARG. This accounts for the typical combinations of RTE products that are consumed by single individuals and also the incidence of AMR found in the measured samples. It is relevant to assess how often there is potential for transmission of AMR genes in the human gut and which ARGs are most prominent. Each individual consumption is linked to the AMR incidence of the combined samples for the relevant food type. This assumption is appropriate when considering the long-term prevalence, because each individual will consume different products over time.

For some individuals, multiple consumptions for the same food type occur within the survey. They may be from the same source and have similar ARG profile or may be from different items. If an individual consumes multiple items with the same ARG present, this is considered the same as if only one of those items contained the ARG. For the purpose of this modelling exercise, it is assumed that there is no aggregation effect (prevalence is proportion of individuals, not proportion of eating events). We also consider the overall population, with many different dietary preferences. Individual subgroups with special dietary habits, e.g., vegetarians, could be analysed separately if the AMR intake for those subgroups was of particular interest.

Suppose we have N individuals i=1,2,…,N in the dietary survey with survey sampling weights $w_{i}$. Consider the food types k=1,2,…,K (K=52) included in the 256 analysed samples. Let j index a particular ARG (j=1,2,…,J). We define the incidence indicator $I_{ijk}$ to be 1 if ARG j is present in food k consumed by individual i and 0 otherwise. Another indicator $C_{ik}$ is set to 1 if food k is consumed by individual i and 0 otherwise. A measure of the total prevalence of ARG j can then be defined as

(1) $$B_{j}=(1/\sum_{i=1}^{N}w_{i})\sum_{i=1}^{N}w_{i}\underset{k}{\max}(I_{ijk}C_{ik})$$

For each ARG, the empirical proportion of all NDNS diets containing the ARG was calculated using Equation (1) to estimate $B_{j}$. Diary survey values for the consumptions of food items were used for $C_{ik}$. Because all individuals are assumed to consume from the same pool of samples over a long time period, $I_{ijk}=I_{jk}$ (same for all individuals), where

$$I_{jk}=\left\{\begin{array}{cc} 1 & 1\;\mathrm{or}\;\mathrm{more}\;\mathrm{samples}\;\mathrm{of}\;\mathrm{food}\;\mathrm{type}\;k\;contains\arg_{j}\\ 0 & \mathrm{otherwise}\; \end{array}\right.$$

An indication of the sampling uncertainty in these estimates is provided by generating 100 bootstrap samples (for each food type, re-sampling with replacement from its original data). The 100 prevalence estimates were used to plot approximate 95% confidence intervals.

These uncertainties do not include measurement uncertainty (i.e., in the absence of an explicit estimate of the false positive and negative rates associated with the identification of ARGs in samples, we assume that the detection of the presence of ARGs is reliable) or sampling uncertainty in the dietary consumption surveys. The latter is expected to be extremely small compared to the sampling uncertainty in ARG measurements, as there are 13,350 individuals surveyed. The numbers of RTE foods sampled are much smaller and often show variability between measurements (e.g., as seen in Section 3.5). The uncertainty intervals are included only to highlight the possible impact of sampling uncertainty. Due to the nature of the intake definition in Equation (1), the true proportions are more likely to be at the upper end of the interval under the assumption of a low false positive rate because increasing the number of samples for a particular food would only lead to higher proportions of diets containing the ARG.

## 3. Results

### 3.1. Sampling Strategy

A sampling strategy was devised such that 90% of UK consumption of RTE foods was covered, with a minimum of five samples per food type. This resulted in 52 different food types being sampled, comprising 33 produce types, 17 dairy types, and 2 cooked meat types. While fewer dairy types were sampled than produce types, more than half of the samples taken were dairy samples. The final list of samples to be taken was distributed across UK regions based on population and purchased from supermarkets based on market share.

### 3.2. Sampling

A total of 1042 samples were collected. Of these, 41 were rejected, leaving a total of 1001 samples to be processed. Samples were rejected for two main reasons: the quality of the product had deteriorated in transit (for example, squashed fruit, leaking ice cream, samples arrived after their use-by date); incorrect sample type had been purchased (for example, semi-skimmed milk rather than skimmed, fat spread of mixed dairy and plant origin).

### 3.3. 16S Sequencing

**Figure 1:**
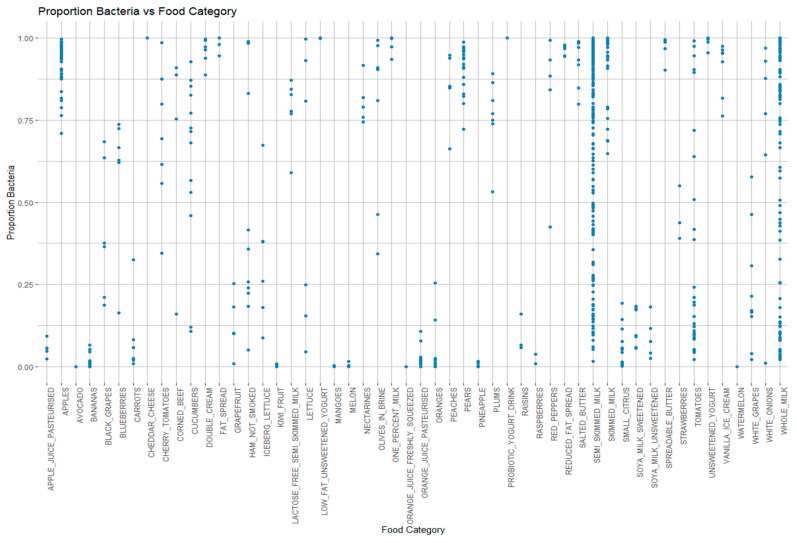
Proportion of bacteria present in each sample, grouped by food category.

The 1001 samples that were analysed by the Qiime2 [23] pipeline were used to identify food categories that had a high proportion of bacteria as opposed to host (chloroplast, mitochondria) sequences. Figure 1 highlights food categories which represent high (e.g., apples) or low (e.g., bananas) proportions of bacteria. Where a range of bacterial proportions are observed within a food category, these were labelled as a continuum (e.g., semi-skimmed milk).

### 3.4. Metagenomic Sequencing

#### 3.4.1. Overview of Metagenomic Read Data

Sequencing summary statistics for NovaSeq sequencing are in Supplementary Information S2.

In total, 8.47 billion raw read pairs were generated from the 256 samples sequenced metagenomically (mean per sample of 33.1 million; median of 32.6 million; see Supplementary Information S2). There were three extreme outliers (the fat spreads), with only 30,336 to 40,747 read pairs. All other samples exceeded 2 million raw read pairs, with 247 in excess of 10 million pairs, of which 153 samples exceeded 30 million. Five samples had more than 50 million, with a highest count of 71,816,590 (semi-skimmed milk sample). In total, 7.87 billion read pairs passed the basic QC, i.e., 92.9% of the raw pairs. Further information on the effects of basic quality control and host-read filtering is provided in Supplementary Information S1.

#### 3.4.2. ARG Sequence Detection Using RGI/CARD

We processed all 256 samples’ quality-controlled, host-filtered unassembled short-read sequence data with the ‘bwt’ mode of the RGI software, which makes use of the associated CARD database and WildCARD [30]. The distribution of read counts means that attempts to assemble the metagenomics reads might have proved fruitful for some samples but would have been inappropriate in the context of the whole study, so the ‘main’ mode of RGI was not used. A total of 1342 unique ARG names occurred in the RGI gene tables; all samples had at least 10 identified. A total of 156 samples had at least 50 ARG names, 101 samples had at least 100, and 27 had at least 300. The highest number of ARG names (548) occurred in the sample (tomato) with the second highest post host-filter read count. The distribution of ARG name counts is shown in Supplementary Information S1 Figure S4. For further information on rates of positive reads and ARGs, see Supplementary Information S1.

For each ARG, RGI reports inferred protein sequence identities with respect to the detected coding gene segments. As multiple read pairs may have matched to each ARG, these are reported as ranges of identities by the software. Using the lower-end identities as a notional worst-case to represent each ARG, we calculated the mean identity as 87%. The median has the same value; although the range is from 20% to 100%, the lower quartile value is 82%.

#### 3.4.3. Filtering of RGI-/CARD-Identified ARGs

While avoiding the most exacting criteria (such as identical sequence matches) for the identification of ARG fragments generally, our aim was to independently identify and discard the lower confidence predictions among the RGI results, since the subsequent modelling made use of presence data rather than frequencies. Therefore, we developed a method which processed the alignment files (SAM/BAM) created by RGI, rather than by using the final RGI tables (which contain metrics of read-to-reference matches averaged per gene or allele within each sample). This pipeline analysed every alignment between reads (pairs) and reference ARG sequences while requiring that each alignment passes multiple quality criteria. After that procedure, 782 different ARG names remained. An additional criterion of 100% sequence identity was imposed only on read matches with ARGs whose phenotype is conferred by small numbers of mutations such as single-nucleotide polymorphisms (“variant/mutant ARGs”). Those ARGs were identified by the appropriate ARO annotation (present for all sequences in CARD), namely, “antibiotic resistant gene variant or mutant”. Finally, we required each ARG to have been identified by at least two independent matches per sample (subsequent to all of the previous filters), so those represented by only a single read pair were removed from that sample’s results. Refer to Section 2 for details.

After applying all of the filters, 477 unique ARG names occurred in the set of 256 samples (counting tet44 and tet(44), which appeared separately but with the same ARO ID (3000556), as the same ARG). See Supplementary Information S1 for further details.

#### 3.4.4. DNA Sequence Identity Distribution

No sequence identity criteria were imposed in our filters (with the exception of the variant/mutant types; see Section 2), but we calculated metrics of the nucleotide sequence identity distribution of all matches remaining after the standard and variant/mutant filters (including the single read pair alignments). For both forward (R1) and reverse (R2) reads passing the filters, the mean and median identities to the reference were 90%. A total of 96% of the passing reads were at least 75% identical to their matching ARG segment, 52% were at least 90% identical, and 29% were at least 98% identical. A total of 20% of reads were 100% identical to their matched ARG. These were calculated strictly as the percentage of the whole (QC) read length that was identical, rather than from a local alignment (the latter would exclude poor-matching segments and thus result in higher average identities). These results are consistent with very high average identity at the protein sequence level (>90%), which is, in turn, consistent with an increase compared to the (pre-filtered) RGI gene tables identities.

#### 3.4.5. Assessing the Use of 16S Metabarcoding for Selecting Samples for Metagenomic Sequencing

A total of 54 samples belonging to food types labelled as ‘low bacteria’ by 16S sequencing (Figure 1) were analysed with Kraken2 [37] to see if these same samples also had low levels of bacteria when sequenced metagenomically. The average percentage of bacterial reads in these 54 samples was 14.4%, ranging from 77.3% to 1.3%, with a median value of 5.6%. Only 5 of these 54 samples had bacterial percentages of greater than 50%. For food types labelled as ‘high bacteria’, the average percentage of bacterial reads was 44.2%, ranging from 99.9% to 1.45%, with a median value of 38.9%. A total of 85 of these samples had bacterial proportions of greater than or equal to 50%.

The 10 samples which had failed 16S sequencing quality control (QC) steps and were selected for metagenomic sequencing were also analysed. Total read numbers from metagenomic sequencing for all 256 samples were ranked from high to low, and the ranks of the 10 ‘failed’ samples were inspected. The median rank was 104, the highest rank was 35, and the lowest rank was 255.

From the above, we can conclude that (A) a sample with low bacterial percentages by 16S sequencing is likely to produce a low number of metagenomic bacterial reads, and (B) samples which fail 16S sequencing QC steps do not necessarily fail when sequenced metagenomically.

### 3.5. Estimation of UK Population Intakes

Following quality filtering, a total of 477 unique ARGs were identified in the RTE samples (see Supplementary Information S3 for a full list of ARGs found in samples). A total of 11 pairs of duplicate samples (same product type, same batch code, bought at the one location during a single visit) were tested for ARGs (Table 2). The aim of the duplicate analyses was to gain a first impression of how consistently representative samples were of the “same product” (product, batch, location, and time of purchase). While we do not expect representative duplicate samples to contain the same ARGs, representative unbiased samples can be expected to contain a similar number of distinct ARGs. The number of ARGs found in each duplicate sample was compared using a Poisson test. Significant values for the Poisson test (blueberries, iceberg lettuce) suggest that factors other than those used to describe the sample are affecting the number of ARGs detected. These may be associated with variation in the product or variation in testing. This may be a topic for further study.

**Figure 2:**
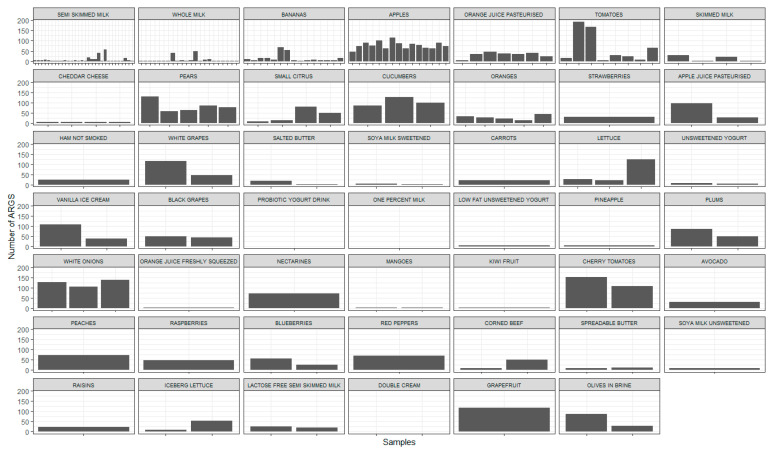
For each sample, the number of unique ARGs found. The x-axis represents unique sample numbers, which have been omitted here to save space. Samples are displayed together per food type, showing the between-sample variation in the number of ARGs. Values are shown for 179 samples (of the original 256 samples, 77 were not found to contain any of the ARGs).

In Figure 2, we see that many produce items have more unique ARGs than dairy products. Some product types have large variation between samples (e.g., bananas, tomatoes, milk). For example, there are two tomato samples (Sample ID = 2664549, 2664550) with 193 and 168 distinct ARGs, whereas other tomato samples have fewer than 50. Pasteurised orange juice, pears, and oranges appear to have similar numbers between samples but have fewer samples. No ARGs were detected in 77 samples., For melon, watermelon, fat spread, and reduced-fat spread, these were the only samples collected, so no ARGs at all were found in these four types. However, the sample sizes were very small in this case (Table 3).

**Figure 3:**
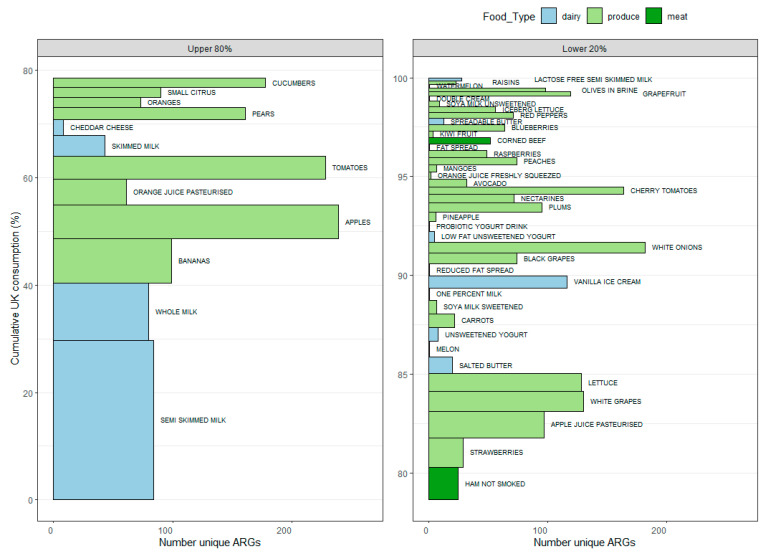
Number of ARGs found per food type. These are ordered by overall consumption.

The number of ARGs found per food type, ordered by consumption and with their cumulative percentage of UK consumption, are shown in Figure 3.

**Figure 4:**
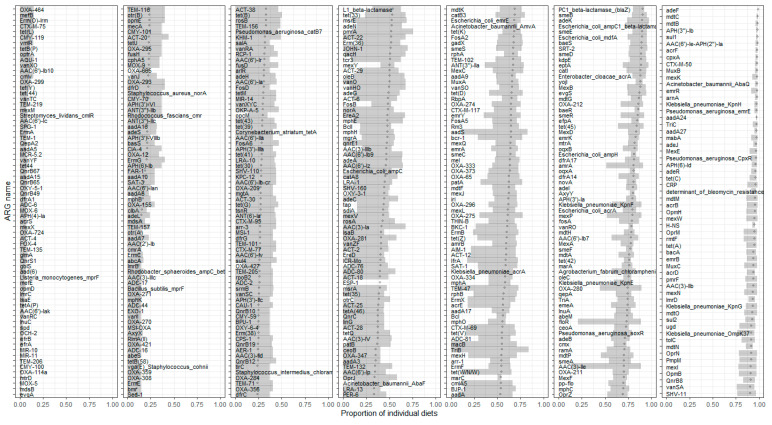
Estimated prevalence of ARGs in ready-to-eat foods, listing those that are estimated to be most prevalent in UK diets. The overall incidences per food were used here. We expect the ARGs found in milk and other high consumption items to be the main contributors with high prevalence due to the frequency of consumption (see, for example, the list of most frequently observed ARGs for semi-skimmed milk). The 95% confidence intervals are shown based on 100 bootstrap samples.

**Figure 5:**
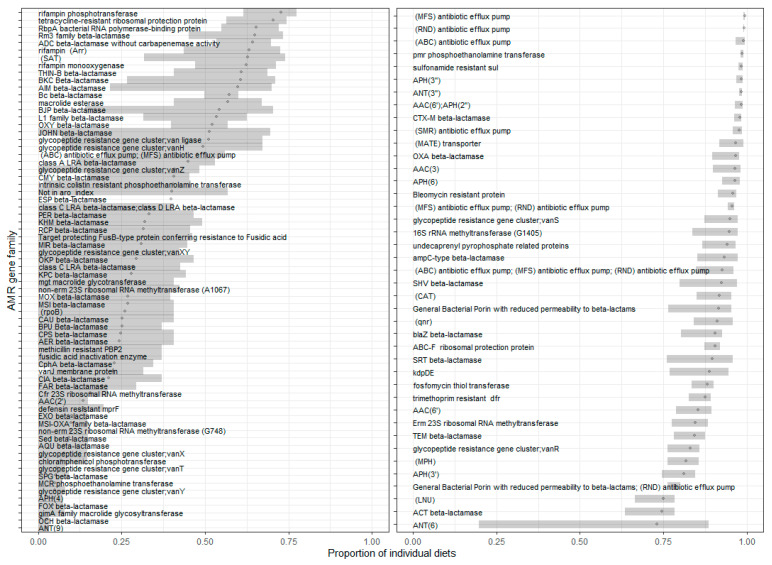
Prevalence (proportion of UK individual diets) for each ARG gene family for all classes found in UK diets. For better readability, particularly given the length of the names on the y-axis, the plots have been divided into roughly equal numbers of points, with panels ordered from highest prevalence at the top to lowest prevalence at the bottom.

The 52 RTE foods sampled in this study are estimated to cover 96.9% of those foods classified as RTE and informative (i.e., excluding foods with multiple ingredients, e.g., sandwiches) within the UK diet (see Section 2). Prevalence estimates for the proportion of individual diets in which individual ARGs and ARG families appear are shown in Figure 4 and Figure 5, respectively.

## 4. Discussion

This study was designed to examine the diversity of ARGs in selected ready-to-eat foods and to estimate the incidence of ARGs in average UK diets. The estimated proportion of individual diets containing the most frequently identified ARGs is extremely high. For example, *adeF*, a gene encoding the membrane fusion protein of the multidrug efflux complex AdeFGH [38], is estimated to be present in approximately 97% of typical diets in the UK. Indeed, the three ARG families found in the highest proportion of diets are all efflux pump families (RND, MFS, and ABC pumps). However, we have insufficient sample numbers to allow for comparison of exposure risk between foodstuffs. As a consequence, despite some food groups appearing to contain more ARGs than others (for example, tomatoes appear to contain more ARGs than semi-skimmed milk in Figure 2), the sample numbers are relatively small, and the difference in ability to detect ARGs on these sample types may be very large. Hence, we deliberately make no comment in this discussion on the different ARG levels present on different food products, although indications of differences between food type may be interesting subjects for further work.

Such high apparent dietary ARG consumption may be driven by several factors. One reason is that DNA sequences generated in this project can be compared against databases containing many hundreds or thousands of different ARG sequences, which is not the case for targeted (e.g., PCR) assays. Hence, it is not surprising that a metagenomic approach should yield relatively high ARG consumption estimates. Furthermore, the metagenomic approach has the potential to detect ARGs in any bacterium on the food sample and even to detect ARGs present in free DNA, unlike phenotypic screening. However, we consider this an advantage rather than a disadvantage of the approach undertaken here. Detection of such ARGs is relevant, as ARGs present as free DNA [39,40,41] or on mobile genetic elements such as bacteriophages [42,43] may be transferred to recipient cells via transformation or transduction. Studies have also demonstrated that transfer of ARGs is also possible after the DNA in question has been heat-treated [40,44,45,46], meaning even detection in cooked or pasteurised foods is worth noting.

Many exercises in the identification of function from sequence data are affected by non-negligible error rates, where some true functions are missed and others are wrongly predicted as present. The prediction of ARGs from sequence data is particularly problematic. Imposing very stringent identity criteria would lead to discarding of many gene fragments whose functional origin might otherwise be predicted with high confidence. Conversely, in some cases, high similarity occurs between ARGs and non-ARGs [34].

The RGI software in general takes a much more sophisticated approach than merely using sequence similarity; however, the ‘bwt’ mode necessarily used for metagenomic short reads employs a protein homology model [30]. We applied filters to the RGI results using quality assessments of the read-to-reference alignments which did not involve sequence identity explicitly, other than for the “mutant or variant” ARG types (where even a single SNP may be the difference between an ARG and non-ARG; we demanded 100% identity for these). These filters aimed to eliminate the lowest-confidence matches, but inevitably, some true positives will have been discarded; at the same time, some false positives will not have been identified as such. However, overall, the resulting high nucleotide sequence identity (average 90%) of the matches passing the filters implies high confidence in the assignments to the reference ARG sequences generally. Aside from the variant mutant types, there are other reasons for the existence of non-AMR genes with high similarity to ARGs, such as common ancestry to proto-resistance genes [34,35]; it is likely that the identified positives will include some such cases.

A separate issue to the faithful identification of ARG reference fragments in the metagenomic data is the integrity of the reference sequences themselves. Some false negatives occur because no ARG database is comprehensive, and false positives may result if the database contains incorrect sequences or annotations. The CARD database, utilised by RGI, is very widely used and a leader in the field; it is intensively curated by experts, with a high evidence threshold for sequence inclusion. Inevitably, it is therefore (relatively) limited in size and likely to be missing many variants. We used the RGI option to include the associated Resistomes, Variants and Prevalence database (WildCARD), previously created by the RGI/CARD developers by processing public sequence data with strict criteria. By its nature, this larger supplement reference cannot be assumed to match the exacting standards of CARD itself, and the false positive rate from matches may be assumed to be greater than if the principal CARD alone has been used. However, given the potentially broad range of bacterial strains that might be encountered in the microbiomes of a large and very diverse set of samples, it was appropriate to include these references in our analysis.

The use of incidence of ARGs as the measure of intakes due to food consumption may also have led to high estimates in this study. Once an ARG is detected on a foodstuff, it is assumed that it will at some point be consumed by any consumer who eats that food type. This is because we did not assume any consumer brand loyalty to a particular sub-type or manufacturer brand, nor did we have sufficient data to capture brand-level variations to link to the NDNS consumption diaries [17].

ARGs which may be involved in resistance to important antibiotics were identified at varying frequencies in the samples (see Supplementary Information S3). Only two colistin resistance ARGs were identified among all samples. *icr-Mo* was found in three food types (apple, nectarine, cherry tomato). *icr-Mo* has so far only been identified in *Moraxella osloensis*, a nematode endosymbiont that has only very rarely been associated with non-foodborne human disease such as bacteraemia [47], endocarditis [48], and meningitis [49]. *icr-Mo* is chromosomally encoded [50] and, as such, is likely of less concern than a mobile colistin resistance gene, as it cannot be easily transmitted to other, more virulent bacteria. Another colistin resistance ARG, *mcr-5.2*, was identified in one cherry tomato sample. *mcr-5.2* is of more interest because it is a mobile (plasmid-borne) colistin resistance gene. *mcr-5.2* was first identified in 2011 in the intestinal contents of a pig in Germany [51]. However, the mcr-5-carrying plasmids identified by Hammerl et al. (2018) did not themselves carry transfer genes involved in plasmid conjugation, meaning other conjugative elements must be present for plasmid transfer [51]. As the current study identified ARGs from short reads, the genomic context of the *mcr-5.2* detected here is not known. The *mcr-5.2* ARG in this study was found in a sample of cherry tomatoes from Morocco, and *mcr* genes have been previously identified in Morocco (and many other countries) [52], but without detailed information about the production processes involved from field to retail sale, it is unknown at what point in production this ARG was introduced. Indeed, a general observation of these results is that the ARGs detected are not necessarily attributable to farming or manufacturing practices. Many ARGs, for example, β-lactamases, evolved for reasons unrelated to clinical or veterinary antibiotic use and have been evolving for millions of years [53]. Such ARGs have been found in pristine habitats, including ancient permafrost [54]. However, anthropogenic factors are known to increase the prevalence of ARGs in particular environments [53].

Methicillin resistance ARGs were also observed very infrequently, i.e., *mecA* in cucumber. The *mecA* gene is found in *Staphylococcus aureus* and encodes a penicillin-binding protein, PBP2’, which is resistant to methicillin [55]. *mecA* is part of the mobile SCC*mec* cassette [56]. Hence, it has the potential to be transferred to methicillin-sensitive *S. aureus*. Its detection is perhaps not surprising, as since its discovery in the 1960s, it has gone on to become globally distributed [56]. The *abcA* gene was also detected, though interrogation of the CARD reference sequence indicates that this sequence originates in the fungus *Aspergillus fumigatus*, and while it may be involved in antimicrobial resistance in the general sense, it is unlikely to be involved in resistance to methicillin.

ARGs which may confer resistance to three other important antibiotics (vancomycin, fluoroquinolones, and carbapenems) were much more frequently detected. Fluoroquinolone resistance ARGs were found in a wide range of foods, particularly fresh produce, including in all samples tested for 37 different food types. This is almost entirely due to various kinds of efflux pump and transporter genes (RND, MFS, MATE, etc.). In fact, the only widely consumed fluoroquinolone resistance ARG family that is not an efflux pump is the quinolone resistance protein (*qnr*).

ARGs that may confer resistance to carbapenems are also found in a high proportion of individual diets. The most common carbapenem resistance gene families, as with fluoroquinolone resistance, are efflux pumps and porins. Although efflux pumps can be plasmid-borne, e.g., [57], chromosomally encoded efflux pumps are common in Gram-negative bacteria [58]. However, in general, efflux pump genes are ancient and widely distributed, usually present in all members of a bacterial species without necessarily indicating resistance to particular antibiotics [59]. Indeed, the high frequency with which efflux pumps were identified highlights another limitation of the techniques deployed here and demonstrates why these measures of ARG presence do not necessarily correspond to presence of phenotypic AMR. Another complication is demonstrated in the case of *van* genes. These may be involved in resistance to vancomycin and work as part of operons [60,61]. The presence of one gene in the operon or gene cluster does not guarantee that the other genes are present or that a vancomycin-resistant phenotype would be observed. The DNA detected could also have originated from viable or inviable intact bacteria at the time of sampling, or as naked environmental DNA (eDNA), which may include complete gene sequences or only shorter fragments. Even ARGs in living bacteria do not necessarily confer a resistance phenotype, as they might not be expressed other than in very specific conditions or only exhibit AMR function when other ARGs are themselves expressed [35]. These are general considerations when working with sequence data rather than phenotypic screening [35]. Considerations regarding potential gene transfer involving all of these states are very complex. Ultimately, the incidence data regards the presence of fragments of genes which, if intact, may have potential for AMR function if expressed in viable bacteria.

The work presented here was ambitious in the breadth of sample types analysed. The methods used in this project were successfully able to detect ARGs in dairy, produce, and meat food types. The surface-rinsing methodology applied to some produce types (see Table 3) appears to have been effective at removing bacterial DNA for sequencing; for some produce (and dairy) samples, high read numbers post-host filtering are obtained. Of the 17 produce types that were surface rinsed, only 2 resulted in low levels of bacteria. Conversely, the 15 produce types that either consisted of juice or were peeled/cut before rinsing resulted in low levels of bacteria in 14 cases. This suggests that, where suitable, surfacing rinsing should be applied in order to obtain more bacterial DNA for sequencing. This is in agreement with data showing that washing with a surfactant is a reliable method for removing bacterial and fungal DNA from apples without the presence of high levels of host DNA [62]. The 16S metabarcoding screen prior to sequencing was effective at identifying samples which had high levels of bacterial DNA relative to host DNA. However, the samples which failed 16S sequencing altogether did not necessarily go on to fail metagenomic sequencing. While this means that some samples may have been screened out unnecessarily, only a low percentage (8%) of samples failed 16S sequencing. It is possible that a measure such as DNA yield may be more appropriate for determining the likelihood of a sample failing metagenomic sequencing, although in this instance, the DNA yield of many samples was below the limit of quantification of the spectrophotometer used, and as such, the maximum input volume of DNA was used.

This work has applied metagenomic sequencing to a wide variety of RTE foodstuffs on retail sale in the UK for the first time. We have demonstrated high levels of ARG consumption, although the link to functional, phenotypic AMR is not known. We provide a baseline against which future surveys can be compared. Our data also suggests the need for methodological improvements (e.g., DNA extraction in foodstuffs that cannot be rinsed). Although we cannot comment on differences in ARG intakes attributed to different food types, a future study structured to address this would be able to build on the results presented here.

## Figures and Tables

**Table 1:** Initial sample processing conditions for produce sample types.

Rinse Outside of Whole Fruit/Slice of Meat or 25 g if Sample Weighs Less e.g., Blueberries (25 mL Rinse Buffer)	Peel and Rinse Whole Interior (25 mL Rinse Buffer)	Cut into with Scalpel and Rinse 25 g Interior Flesh	Juice Centrifuge Sample Directly
Apples	Banana	Melon	Orange Juice 1 mL
Pears	Orange	Watermelon	Apple Juice 15 mL
Nectarines	Small citrus (e.g., satsuma/mandarin/clementine)	Pineapple	
Peaches	White onion	Kiwi	
Plums	Grapefruit—used 50 mL rinse buffer	Mango	
Strawberries	Carrot	Avocado	
Blueberries			
Raspberries			
Cherry tomatoes/Tomatoes			
Cucumber—used 50 mL rinse buffer			
Lettuce			
Red pepper			
White/black grapes			
Ham			
Corned beef			
Raisins			
Olives			

**Table 2:** Food categories for which duplicate samples were tested, showing the number of ARGs identified in each sample, the number shared in both samples, and the total number of different ARGs found across the pair of samples. Poisson’s test (adjusted for multiple comparisons) significance values are shown.

Food Category	ARGs in Each Sample	Number in Both Samples	Number in Either or Both Samples	Poisson Test
apples	85, 79	56	108	1
blueberries	57, 25	18	64	0.00535
cherry tomatoes	154, 109	99	164	0.059
double cream	1, 1	1	1	1
iceberg lettuce	9, 55	8	56	3.89 × 10^−8^
lactose-free semi-skimmed milk	25, 21	18	28	1
oranges	27, 21	14	34	1
spreadable butter	10, 11	8	13	1
tomatoes	193, 168	149	212	1
unsweetened yogurt	7, 5	4	8	1
white onions	107, 139	94	152	0.383

**Table 3:** Summaries based on the number of ARGs per sample, by food type. The number of samples collected and the number of samples containing at least 1 ARG are also shown.

Food Category	Summary Based on Number of ARGs Per Sample				Number of Samples	
	Mean	Median	Min	Max	Total Samples	Samples with 1 or More ARG
semi-skimmed milk	2.9	0	0	56	69	30
whole milk	3.3	1	0	49	42	22
bananas	13.5	5.5	0	68	16	15
apples	78.1	76	47	114	15	15
orange juice pasteurised	31.3	36	3	45	7	7
tomatoes	63.0	26	3	193	8	8
skimmed milk	7.6	1	0	30	7	4
cheddar cheese	5.8	6	5	6	4	4
pears	82.4	76	58	129	5	5
small citrus	36.8	30	7	80	4	4
cucumbers	102.7	98	84	126	3	3
oranges	27.2	27	12	44	5	5
strawberries	29.0	29	29	29	1	1
apple juice pasteurised	61.5	61.5	27	96	2	2
ham not smoked	6.0	0	0	24	4	1
white grapes	80.5	80.5	46	115	2	2
melon	0.0	0	0	0	1	0
salted butter	10.5	10.5	3	18	2	2
soya milk sweetened	4.5	4.5	3	6	2	2
carrots	22.0	22	22	22	1	1
lettuce	57.7	27	21	125	3	3
unsweetened yoghurt	6.0	6	5	7	2	2
vanilla ice cream	74.0	74	39	109	2	2
black grapes	47.0	47	43	51	2	2
reduced-fat spread	0.0	0	0	0	2	0
probiotic yoghurt drink	1.0	1	1	1	1	1
one-percent milk	1.0	1	1	1	1	1
low-fat unsweetened yoghurt	5.0	5	5	5	1	1
pineapple	6.0	6	6	6	1	1
plums	68.0	68	51	85	2	2
white onions	124.7	128	107	139	3	3
orange juice freshly squeezed	2.0	2	2	2	1	1
nectarines	72.0	72	72	72	1	1
mangoes	2.3	3	0	4	3	2
kiwi fruit	0.8	0	0	3	4	1
cherry tomatoes	131.5	131.5	109	154	2	2
fat spread	0.0	0	0	0	1	0
avocado	10.3	0	0	31	3	1
peaches	74.0	74	74	74	1	1
raspberries	49.0	49	49	49	1	1
blueberries	41.0	41	25	57	2	2
red peppers	71.0	71	71	71	1	1
corned beef	29.0	29	8	50	2	2
spreadable butter	10.5	10.5	10	11	2	2
soya milk unsweetened	9.0	9	9	9	1	1
raisins	23.0	23	23	23	1	1
iceberg lettuce	32.0	32	9	55	2	2
lactose-free semi-skimmed milk	23.0	23	21	25	2	2
watermelon	0.0	0	0	0	1	0
double cream	1.0	1	1	1	2	2
grapefruit	119.0	119	119	119	1	1
olives in brine	58.5	58.5	29	88	2	2

## Data Availability

Sequences have been deposited in the European Nucleotide Archive under accession ERP128088. Code is available at https://gitlab.fera.co.uk/jwalshaw/argisamfilter/, accessed on 22 May 2025.

## Supplementary Materials

The following supporting information can be downloaded at: https://www.mdpi.com/article/10.3390/microorganisms13081766/s1, Supplementary Information S1: Basic NovaSeq sequence data Quality Control methods; Supplementary Information S2: Sequencing summary statistics for NovaSeq sequencing; Supplementary Information S3: Full list of ARGs found in samples; Supplementary Information S4: Reference genomes used for host sequence removal; Supplementary Information S5: Quality-control of metagenomics short-reads aligned to reference ARG sequence database; Supplementary Information S6: Technical notes on metagenomic short-read analysis.

## Abbreviations

AMR	antimicrobial resistance
ARG	antimicrobial resistance gene
ARO	antibiotic resistance ontology
ASV	amplicon sequence variant
BAM	binary alignment map
bp	basepair
CARD	Comprehensive Antibiotic Resistance Database
CIA	critically important antimicrobial
DNA	deoxyribonucleic acid
dNTP	deoxynucleoside triphosphates
dsDNA	double-stranded deoxyribonucleic acid
eDNA	environmental DNA
NDNS	national diet and nutrition survey
PCR	polymerase chain reaction
RGI	resistance gene identifier
RTE	ready-to-eat
SAM	sequence alignment map
TE	tris-ethylenediaminetetraacetic acid
UK	United Kingdom
