# Supplementary material for: Metagenomic Analysis of Ready-to-Eat Foods on Retail Sale in the UK Identifies Diverse Genes Related to Antimicrobial Resistance
Source: Microorganisms. 2025 Jul 29;13(8):1766. doi: 10.3390/microorganisms13081766 (PMC12388329; doi:10.3390/microorganisms13081766)
Supplement: Supplementary file 1 [file microorganisms-13-01766-s001.zip › Supplementary Information S1.pdf]

Basic quality control (QC)

In total, 7.87 billion read pairs passed the basic QC, i.e. 92.9% of the raw pairs; 234 of the 256 samples had a pass-rate ≥ 90%. Four outlier samples (iceberg lettuce, corned beef, apple juice pasteurised, bananas) had a pass-rate of 62% - 78% of read pairs, but were still left with between 12 million to 30 million pairs post-QC.

The number of post-QC reads in each sample can be seen in Figure S1 (x-axis).

Host-read filtering

The "host" is the food organism, whose DNA sequences may be present in the metagenomic data.

The numbers of read pairs remaining in each sample after host-filtering (absolute values, and as a percentage of the post-QC counts) is provided in Supplementary Information 2.

Figure S1 shows the post host-filtering number of reads versus the original input (i.e. post-QC) number of reads for each sample.

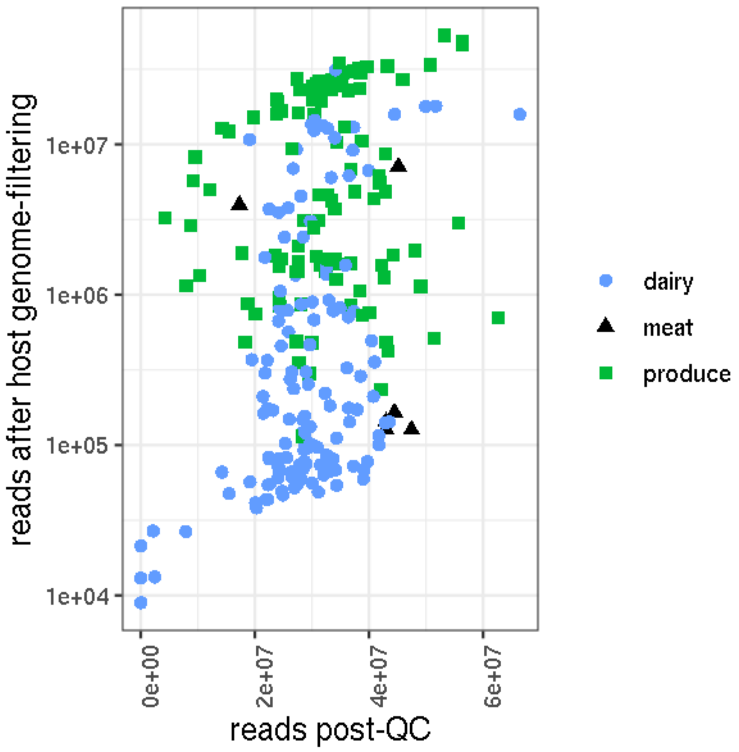

Figure S1. Read counts passing basic QC and subsequent host-DNA filters. Each point represents one sample

Where few reads were removed at the host-filtering stage, this is due either to the original sample containing few host organism reads, or to difficulties with host-read detection (due to reference genome sequence relatedness or quality, for example).

In general, fewer host reads were detected and removed from the produce type than from dairy or meat. The profile was generally consistent within individual food categories, notwithstanding that some categories are represented by a very small number of samples (Figure S2, Figure S3).

Many samples had a very low pass rate, i.e. a very high rate of identified host read pairs. 90 samples had < 1% of post-QC read pairs remaining after host-filtering, numbering 13,000-420,000 read pairs, with 37 of these samples numbering > 100,000 pairs. The absolute counts are the most pertinent in

terms of utility of the metagenomics data (y-axis of Figure S1), given that some samples (fat spreads) had a good pass rate but had very few reads to begin with. Of the 256 samples, after host-filtering one sample yielded < 10,000 read pairs, with a total of 57 samples < 100,000, and 109 samples < 500,000 pairs. 125 samples exceeded 1 million read pairs, of which 12 exceeded 30 million. The highest count was over 53 million (white onion).

The distribution is not unimodal and notably reflects different trends among the food types. Of the 30 samples with > 20 million remaining reads, all but one (probiotic yoghurt drink) are of 'produce' type. Conversely, of the 100 samples with the lowest post host-filtering read counts, only four are produce samples (watermelon and three mango), four are the meat samples and the remainder are dairy. Profiles of individual food categories highlight contrasts within the broader food-type (Figure S2, Figure S3). For example, the two spreadable butter samples (among dairy, the 3rd and 4th highest post-host-filter read counts) have similar host-filter rates to the fat spreads, but these two categories are at opposite ends of the final dairy read-count distribution.

A high proportion of host sequences among the sample DNA is not unexpected in some categories such as milk, of which only three samples returned more than 10 million read pairs post host-filter (16 milk samples exceeded 1 million pairs). 54 milk samples (of total 124) had fewer than 100,000 pairs remaining.

Effect of filtering RGI/CARD-identified ARGs

After applying all filters, 77 samples had no ARGs detected, 28 had only a single ARG name and 10 or fewer ARGs were detected in 159 samples. 49 samples yielded 50 or more and 15 samples at least 100 ARG names (the maximum of 193 occurred in the same tomato sample previously referred to). Compared to the original RGI results (Figure S4), most ARGs were discarded as a result of the standard filters applied to each read-reference alignment (Figure S5). The additional filter applied to the variant/mutant ARGs had a much smaller but still tangible effect; of the 779 ARG names remaining, the discarding of single read-pair ARGs removed a further 301.

Effect of the variant/mutant-type filter

Of the 782 unique ARG names which remained after the standard filter, only 6 were of the variant/mutant type (ARO term 0000031); Table S1.. Two of those were very similar names for the same gene. These 6 consisted of 3 *rpoB* genes and *ileS, parY* and *soxR* from various species. At least one of these occurred in 189 samples, with incidence of any one ranging from 23 to 147 samples. After applying the 100% match identity filter, three of these ARGs were eliminated from all samples, with the others present in 1, 3 and 33 samples. After applying the final filter requiring at least two remaining read pairs per ARG per sample, only two ARGs remained: *rpoB* positive in only one sample (represented by 2 read pairs only), and *Pseudomonas aeruginosa soxR* positive in 17 samples (between 2 and 18 read pairs). These results are consistent with most or all of the original detections of these genes being false positives. Not all false positives (sequence reads originating from the wild-type or other non-AMR variant of the gene) would be removed because some reads would correspond to segments which were identical in the ARGs and non-ARGs.

Rates of positive reads and ARGs

Given the very broad range of read counts that were input into the ARG sequence-detection stage, the absolute numbers of ARG names and indeed ARG-positive read pairs were inevitably related to the input read counts. However, the proportion of ARG-positive read pairs is not correlated to the input read counts (Figure S6); this result is consistent with a widely-differing number of true ARG DNA

fragments in the samples, coupled with an approximately proportional detection rate. This is also consistent with the rate of ARG detection being not well-predicted by the input read-pair count, albeit loosely inversely correlated (Figure S7). This correlation is expected if there is a notional maximum number of unique ARGs that may be present (or at least, detectable by the methods and database used) in a sample; increasing sequencing depth would thus yield decreasing numbers of ARG per read pair.

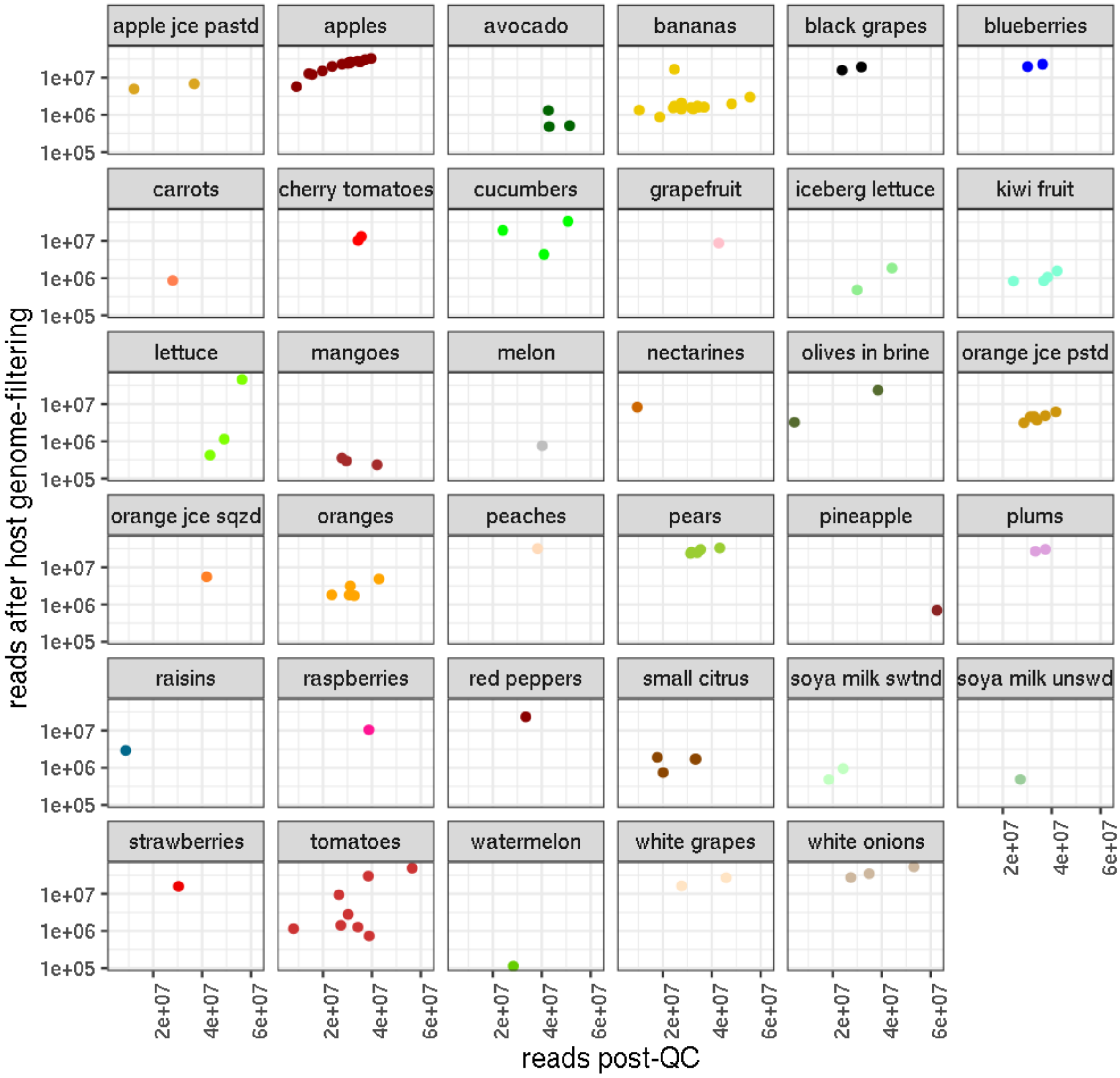

Figure S2. Read counts passing basic QC and subsequent host-DNA filters. Each point represents one sample in the produce food category. Abbreviations "appl jce pastd", "orange jce pstd", orange jce sqzd", "soya milk swtnd", "soya milk unswd" respectively represent the names

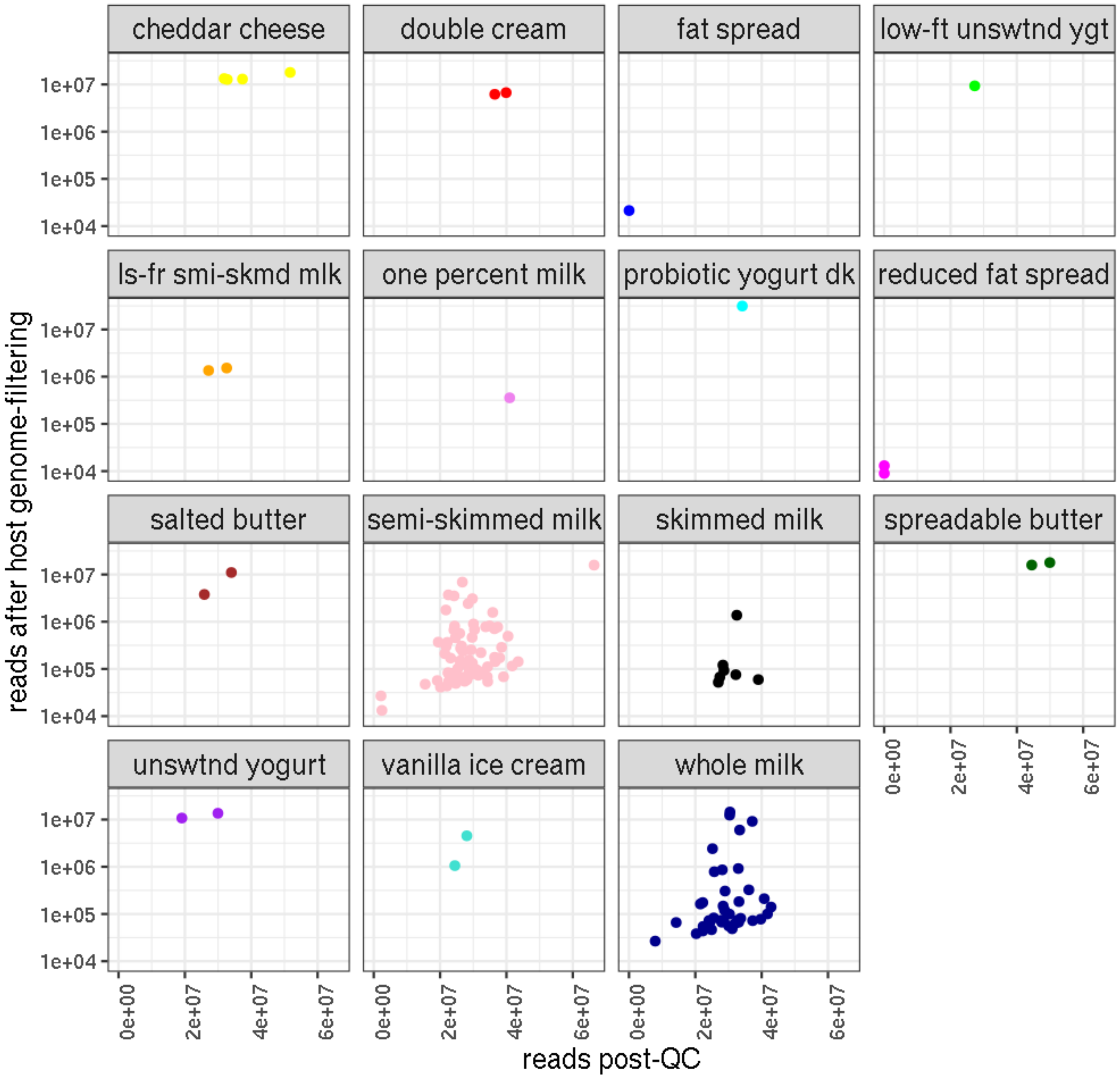

Figure S3. Read counts passing basic QC and subsequent host-DNA filters. Each point represents one sample in the dairy food category.

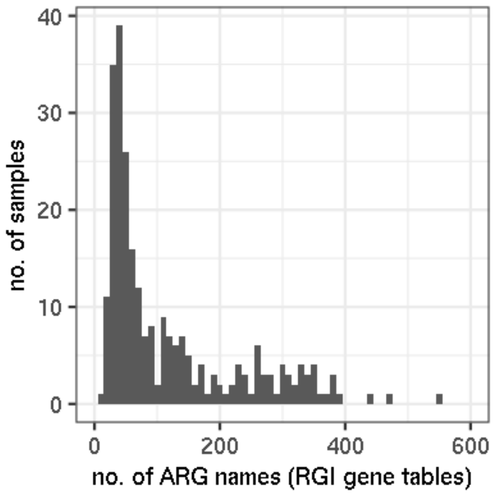

Figure S4. Numbers of unique ARG names in the gene tables (RGI bwt-mode output).

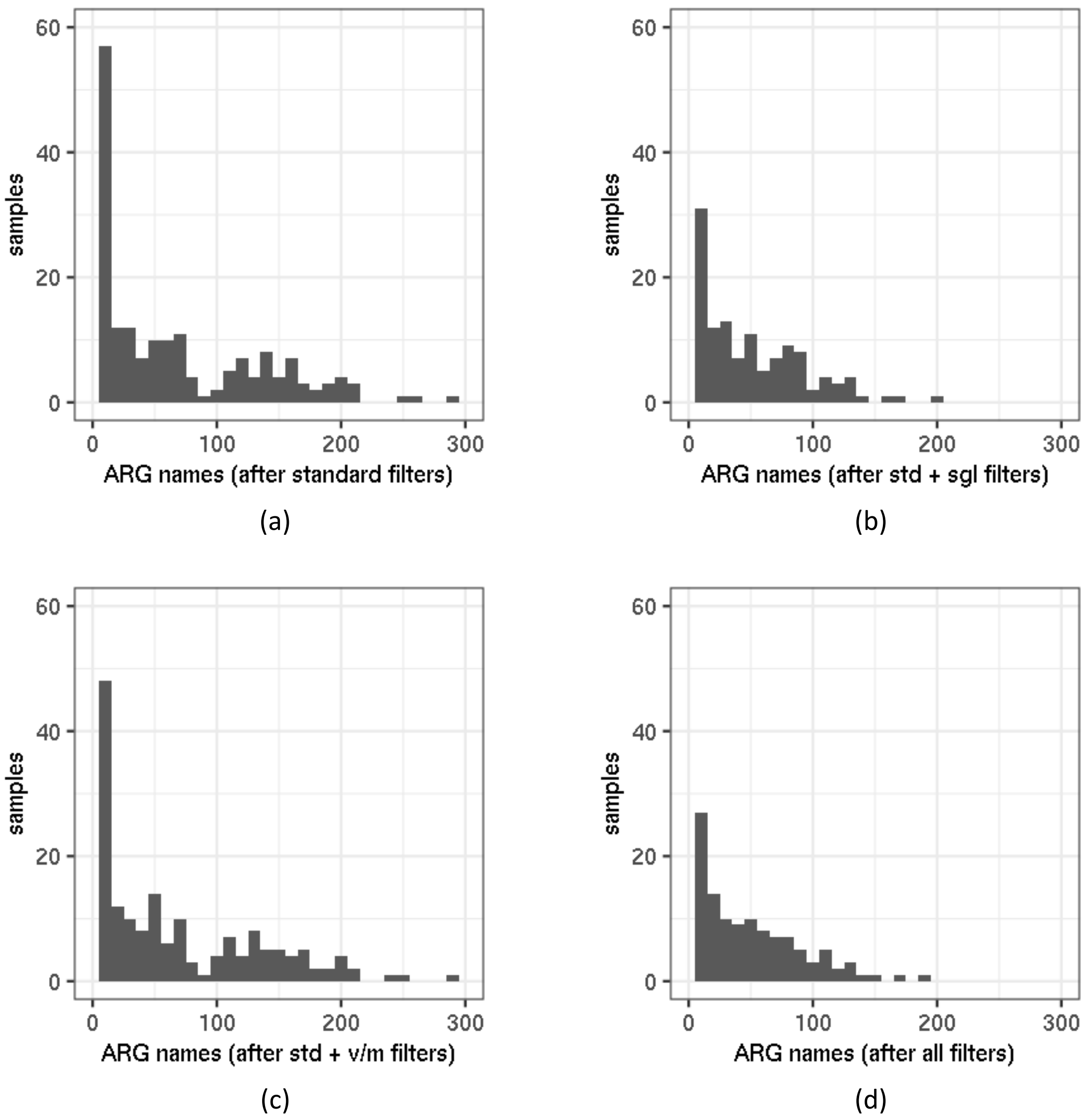

Figure S5. Numbers of unique ARG names remaining after applying filters to the reads-to-references alignments (RGI bwt-mode SAM files). (a) Frequencies after the standard filters applied to all alignments. (b) As (a), additionally with subsequent removal of all ARGs represented by only a single remaining read pair in each sample. (c) As (a), additionally with subsequent removal of all instances of 'variant/mutant' type ARGs matches where the alignment identity was not 100%. (d) As (c), additionally with subsequent removal of all ARGs represented by only a single remaining read pair in each sample; this represents the final ARG identifications used for the modelling. As described in Methods, the standard production flow is (a) → (c) → (d); the results of (b) were calculated here to assess relative contributions of each filter.

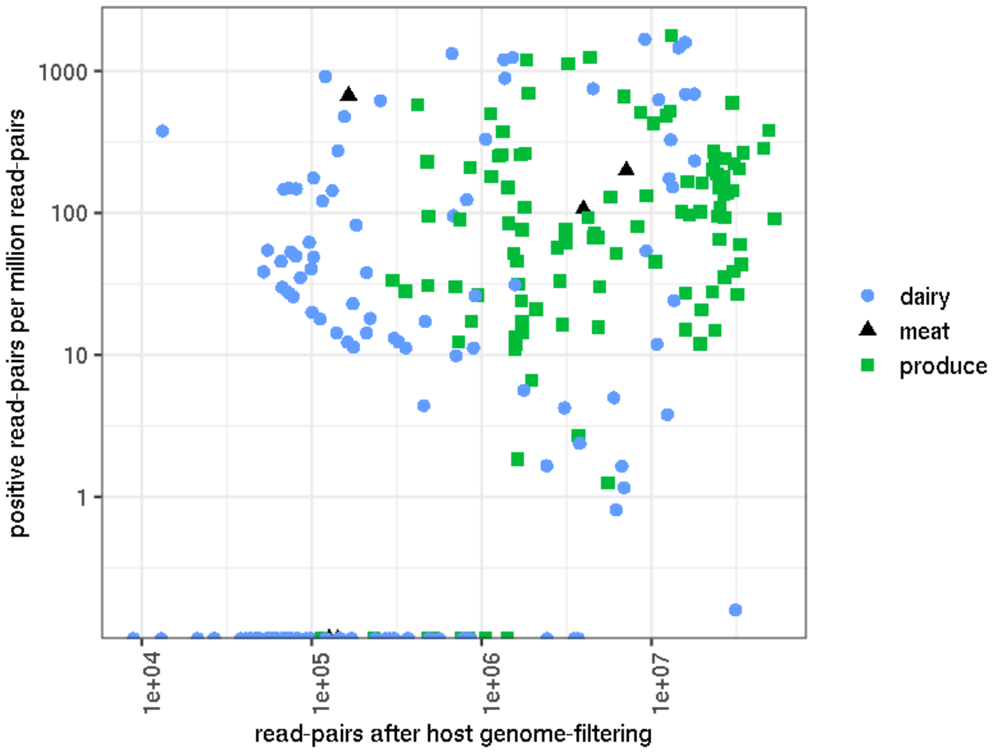

Figure S6. The proportion of read-pairs which make a positive ARG match (after all filters have been applied). Each point represents one sample. 77 samples have 0 positive read-pairs. Due to the final stage of filtering, the lowest possible non-zero number of positive read-pairs is 2, which occurs in 11 samples, visible as the straight diagonal line (different values of 2/(*N* input read-pairs) on the log scales).

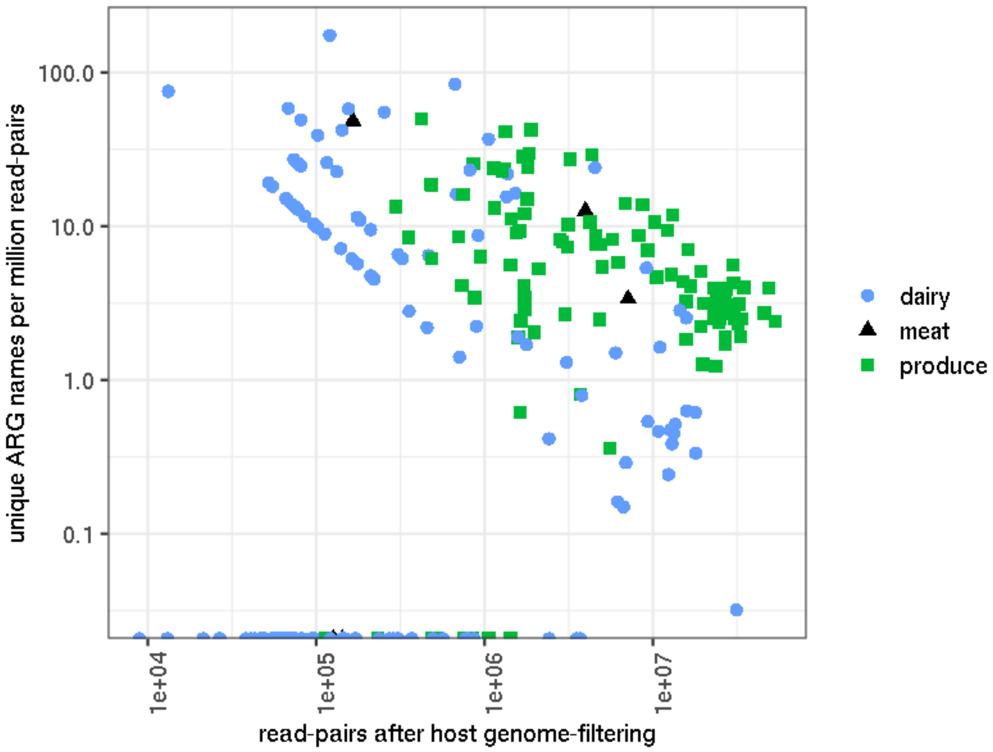

Figure S7. The rate of identification of unique ARG names per input read pair (after all filters have been applied). Each point represents one sample. 77 samples have 0 identified ARGs. 28 samples have 1 identified ARG, 11 have 2 and 15 have 3, resulting in the diagonal lines (different values of *x*/(*N* input read-pairs) on the log scales).

Table S1. Incidence of the ARGs of type "antibiotic resistant gene variant or mutant" (ARO:0000031) in the samples following the standard filter, and subsequently after the VM filter (matches to variant/mutant-type ARGs must have 100% nucleotide sequence identity); and finally after the read-pair count filter (elimination of ARGs from a sample where < 2 read pairs passed the previous filters). *Two very similar ARG names were present in the reference databases for two sequence variants of ARO:3004480.

ARG name	ARO accession	number of positive samples after standard filter		
		without additional VM-filter	with additional VM-filter	with additional VM and minimum 2-prs filters
Bifidobacterium adolescentis rpoB conferring resistance to rifampicin*	3004480	23	0	0
Bifidobacterium adolescentis rpoB mutants conferring resistance to rifampicin*	3004480	147	1	0
rpoB2	3000501	122	3	1
Bifidobacterium ileS conferring resistance to mupirocin	3003730	87	0	0
Streptomyces rishiriensis parY mutant conferring resistance to aminocoumarin	3003318	79	0	0
Pseudomonas aeruginosa soxR	3004107	72	33	17
one or more of the above		189	35	18
