# Supplementary material for: Metagenomic Analysis of Ready-to-Eat Foods on Retail Sale in the UK Identifies Diverse Genes Related to Antimicrobial Resistance
Source: Microorganisms. 2025 Jul 29;13(8):1766. doi: 10.3390/microorganisms13081766 (PMC12388329; doi:10.3390/microorganisms13081766)
Supplement: Supplementary file 1 [file microorganisms-13-01766-s001.zip › Supplementary Information S5.pdf]

Quality-control of metagenomics short-reads aligned to reference ARG sequence database

To process short-read metagenomics data, the RGI software was run in 'BWT' mode, independently on each sample's reads. This produces a tabular format, where each column contains various metrics describing the quality of the matches between the reads and the AMR genes (ARGs). Each row represents one ARG; this means that the metrics are averages over all the reads in the sample matched to that ARG. This is of limited utility for our purposes, and so we have processed the RGI BWT output in a different way.

Here, we briefly describe the input data, the default tabular output data and other output produced by RGI BWT, to clarify how we have processed this data, since this involves our own post-processing of the RGI output which does not occur with normal use of this software.

Summary of RGI BWT inputs and outputs

Each sample's metagenomics sequence data is in the form of a FASTQ-format file of the paired-end (**PE sequence**) reads (which have previously been subject to quality-filtering and removal of host-origin sequences). RGI BWT uses the **BowTie2** "mapping" software to align the read-pairs against matching sequences (if any) in the **CARD** database of ARG sequences. The immediate output is alignment file in **SAM format** (The SAM/BAM Format Specification Working Group, 2021). This is then automatically further processed by RGI BWT to determine numerous metrics, principally the numbers of reads (read pairs) which have been mapped to each ARG, and also statistics summarising the quality of those matches. Thus two final tabular outputs, the **ARG gene table** and **ARG allele table**, are output. (Figure S8).

In each row of the **ARG gene table** for a sample, the number of matching reads is specified in various contexts (such as wholly-matching and partially-matching). This effectively provides relative quantitative information, within the sample. Various other columns state some ARG-specific attributes (cross-references to ARO accessions, etc) and metrics describing the matches between the reads/read pairs and the ARG reference sequence (such as average coverage of the reference sequence in terms of percentage length and average length; average MAPQ score of completely-matching reads; etc).

These various per-read (or per-read pair) quality metrics are necessarily *averaged*, since there is no *per-read* data. Therefore, individual read ↔ ARG reference matches cannot be inspected, and nor can they be screened further for the purpose of additional quality control.

A similar table, the **ARG allele table**, is at the finer granularity of individual allelic variants of each ARG.

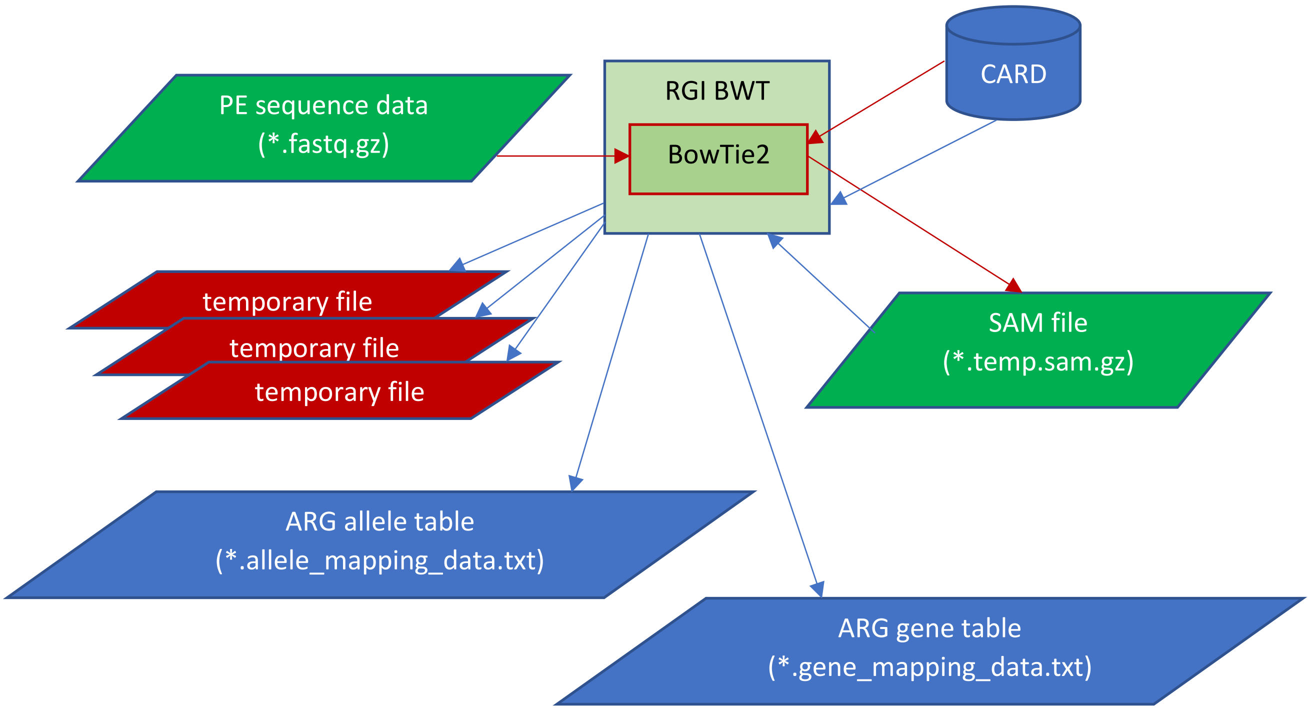

Figure S8. RGI BWT processing, inputs and outputs. Files are represented by parallelograms, databases by cylinders and processes by rectangles. The green files are inputs used at various stages by RGI BWT (the SAM file is an intermediate file created by BowTie2 which is run by RGI BWT). The standard output table files are in blue. Many temporary files of no further relevance to our post-processing are also created by RGI BWT (red), in the course of its processing the read data of one sample.

Temporary/intermediate files created by RGI BWT

In the normal, "clean" mode, the SAM file, along with many other temporary files created during the processing, are deleted on completion, leaving only the output tables (as well as a small number of other brief files which summarise total number of reads processed and the numbers which could be matched to any ARGs).

However, if run in "noclean" mode, numerous intermediate working files remain on completion, as well as the SAM-format alignment file, created by the short read-mapping software BowTie2 (which is run automatically as part of the execution of RGI BWT), also depicted in Figure S8. Since this file contains details for each read that is mapped to (matched with) a reference ARG sequence, this can be used to assess the details of each and every alignment of a read (pair) to an ARG sequence. In our post-processing procedure, we analyse all of the alignments in the SAM files and delete the other temporary files.

Assessment of short-read metagenomics sequences matched to reference ARGs

The principal aim of the assessment is to identify **likely false-positive** matches and discard them. By its nature, short-read data will have a higher rate of false matches (or conversely, false absence of matches) than complete genomic data. A false positive is where the sequence read (pair) makes a good match to a reference ARG despite the read not originating from that gene. **False negatives** represent instances of (i) ARGs being present in the sample, but not generating any sequence reads; or (ii) generating sequence reads which are not matched to the reference sequence. Type (i) is by

definition not possible to assess from the sequence data; type (ii) would require a re-assessment of all of the reads, which is beyond the scope of this analysis.

Assessing causes of identifiable false positives

Attributes of the read ↔ reference mapping to be considered can be broadly categorised as follows:

- Lengths of matching read segments and any mismatching segments
- Uniqueness of the mapping
- Plausibility of the sequences themselves irrespective of whether the read and reference sequences are very similar
- Sequence identity of the matching read segments.

A key part of the assessment of each read ↔ ARG match is to determine what proportion of the read makes a good match with the reference sequence, and what proportion fails to match. A segment of the read failing to match is perfectly acceptable if the reason is that it extends beyond the end of the reference sequence, but otherwise it is a sign that the overall matching of the read with the reference is not reliable. As well as the proportions, the absolute length of the well-matching segment must be considered.

In Figure S9, the sequence read (depicted variously in green, yellow, orange) is shown on top, with the reference ARG sequence on the bottom (blue).

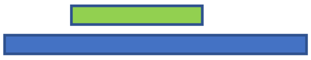

Figure S9(a). Fully aligned to reference; no part of the read has been clipped off. Unless this is a very short read, i.e. shorter than the expected minimum length, then it will be retained in this part of the screening.

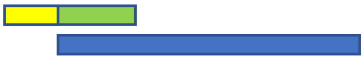

Figure S9(b). Partially aligned to reference; the part of the read overhanging the 5' end of the reference has been clipped off. If the remaining (green) part of the read is sufficiently long, it will be retained in this part of the screening. No further consideration is made of the clipped (yellow) segment, regarding sequence identities, etc.

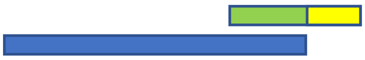

Figure S9(c). Partially aligned to reference; the part of the read overhanging the 3' end of the reference has been clipped off. The same principles apply as for the 5'-overhangs.

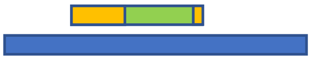

Figure S9(d). Partially aligned to reference; part(s) of the read (orange) have been clipped off from either (or both, as in this case) of the 5' and 3' ends of the read, because the match is too poor. Unless

the clipped segments are very short, this indicates that this read makes a dubious alignment with this reference overall, and should be **discarded**.

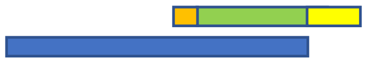

Figure S9(e). Partially aligned to reference; as before, the overhanging part of the read (yellow) is not considered in the evaluation. One end of the read (orange) has been clipped off from the 5' end, because the match is too poor. Again, if the clipped segment is too long, the read will be discarded, irrespective of the aligned segment (green). If the clipped segment is short enough, then the read will still be discarded if it constitutes too large a proportion of the total **non-overhanging** part (green plus orange).
