# Supplementary material for: Metagenomic Analysis of Ready-to-Eat Foods on Retail Sale in the UK Identifies Diverse Genes Related to Antimicrobial Resistance
Source: Microorganisms. 2025 Jul 29;13(8):1766. doi: 10.3390/microorganisms13081766 (PMC12388329; doi:10.3390/microorganisms13081766)
Supplement: Supplementary file 1 [file microorganisms-13-01766-s001.zip › Supplementary Information S6.pdf]

Supplementary Information file 6: Technical notes on metagenomic short-read analysis.

Quality-control (trimming)

Summary statistics for each sample were generated using fastqc and multiqc, before low quality sequences were trimmed using sickle in paired-end mode, with a minimum quality score of 20, a minimum length of 50 and no 5-prime trimming parameters set.

Detection and removal of host DNA-origin reads

Each sample was mapped against a relevant host genome using bwa mem with default parameters in paired-end mode. The subsequent .sam file was filtered and unmapped read IDs were identified using the read unmapped flag. Unmapped reads were then extracted using the seqtk subseq command, with the unmapped read IDs as input.

Analysis with RGI

We downloaded RGI (version 5.1.0) and CARD (3.0.8) and associated Resistomes, Variants and Prevalence data ("WildCARD", 3.0.6), on 11th May 2020.

Initialising RGI

The CARD/WildCARD databases were loaded into RGI, following the RGI documentation. It is assumed that shell variables CARDdata and WILDCARDdata have been set to the paths containing the CARD and WildCARD data files (uncompressed) respectively; and that a local-level (not system-level) installation of the databases is employed.

Firstly, the CARD database was loaded and annotated with the following commands:

```
rgi load --card_json $CARDdata/card.json --local

rgi card_annotation -i $CARDdata/card.json
```

(generated file `card_database_v3.0.8.fasta`).

The same procedure was followed for WildCARD:

```
rgi load -i $CARDdata/card.json \
    --card_annotation card_database_v3.0.8.fasta --local

rgi wildcard_annotation -i $WILDCARDdata \
    --card_json $CARDdata/card.json -v 3.0.6
```

(generated file `wildcard_database_v3.0.6.fasta`)

The annotations were then loaded with the databases into RGI:

```
rgi load --wildcard_annotation wildcard_database_v3.0.6.fasta \
    --wildcard_index $WILDCARD_data/index-for-model-sequences.txt \
    --card_annotation card_database_v3.0.8.fasta --local

rgi load --kmer_database wildcard_uncompressed/61_kmer_db.json \
```

```
--amr_kmers $WILDCARDdata/all_amr_61mers.txt --kmer_size 61 \
--local
```

These commands will create a directory `localDB/` in which files of various formats will have been created. Among these is `card_wildcard_reference.fasta`, referred to later.

Running RGI

RGI was applied to each sample with a command of the form:

```
rgi bwt --read_one path_to_reads/sampleID_R1.fq \
        --read_two path_to_reads/sampleID_R2.fq \
        --aligner bowtie2 \
        --output_file \
            data/rgi_output/rgi_bwt_sampleID/rgi_bwt_sampleID \
        --threads 32 --include_wildcard --local
```

For example, for the sample with ID 6400, this creates, in the output parent directory `data/rgi_output/`, a subdirectory named `rgi_bwt_6400/` containing the output files which have a prefix 'rgi_bwt_6400.':

```
rgi_bwt_6400.allele_mapping_data.json
rgi_bwt_6400.allele_mapping_data.txt
rgi_bwt_6400.artifacts_mapping_stats.txt
rgi_bwt_6400.coverage_all_positions.summary.temp.txt
rgi_bwt_6400.coverage_all_positions.temp.txt
rgi_bwt_6400.coverage.temp.txt
rgi_bwt_6400.gene_mapping_data.txt
rgi_bwt_6400.model_species_data_type.temp.txt
rgi_bwt_6400.overall_mapping_stats.txt
rgi_bwt_6400.reference_mapping_stats.txt
rgi_bwt_6400.rgibwt.stderr
rgi_bwt_6400.rgibwt.stdout
rgi_bwt_6400.seqs.temp.txt
rgi_bwt_6400.sorted.length_100.bam
rgi_bwt_6400.sorted.length_100.bam.bai
rgi_bwt_6400.sorted.temp.bam.bai
rgi_bwt_6400.sorted.temp.bam
rgi_bwt_6400.temp.bam
rgi_bwt_6400.temp.sam
rgi_bwt_6400.temp.txt
```

The gene and allele tables (text-format) referred to in the manuscript are highlighted in bold, as is the SAM file used in downstream analysis. For that reason, the `--clean` option was not used for `rgi bwt` (it deletes the *.temp.* files when the processing is complete).

The command was run on the pair or reads files for each of the 256 samples, totalling 245 CPU hours on several 80 x 2.4 GHz or 144 x 2.5 GHz CPU core Linux servers, via a Nextflow script (`rgi_bwt_from_list.nf`; see *Pipeline* section below).

Filtering the RGI results by alignment properties

The filtering of the ARGs identified by RGI bwt (screening the read-to-reference alignments to discard those with characteristics most consistent with false positives) consisted of several stages (refer to manuscript Methods section for details):

1. standard filter (applied to all alignments; consists of multiple steps)
2. 'variant/mutant' filter (100%-identity screen applied only to alignments with ARGs annotated with Antibiotic Resistance Ontology term ARO:0000031 or one of its descendent terms; thus a 'term-specific' screen)
3. read-pair count screen (per ARG per sample)

The standard first stage was implemented as three R scripts, and the term-specific second stage with three further R scripts and two Perl scripts. (Refer also to the *Pipeline* section.) The final stage was a trivial filter to remove rows from a data table which have a value of < 2 in the appropriate integer data column. The R scripts were run using R 4.0.1 and require the stringr and plyr libraries.

1) Standard filter

This stage consisted of several R scripts: one reads the SAM-format data and calculates various properties of the alignment as described in Methods (main manuscript) and outputs a tabular format data file where each row represents a read pair; the second applies filters to several of the properties, in turn, to each individual read; one of the metrics is a combined property of both reads, i.e. the sum of the matched segments. The third script creates a tabular format file where each row represents one ARG.

The stages of the standard filter are implemented in the Nextflow scripts `reshape_rgi_bwt_sam.nf` and `scrn_wrt_sqs_rgi_bwt_pairs.nf` - the latter also implements some stages of the next filter (refer to the *Pipeline* section), regarding the assessment of properties of independent alignments of the reads to the reference ARG sequences.

2) Variant/mutant filter (ARO term-specific screen)

This filter (removal of reads whose identity to their matched ARG sequences < 100%) applies conditionally to particular ARGs only. Therefore, this stage requires a reference data file specifying whether or not each ARG is of the type appropriate for this filter. Since the percentage identity is assessed by an independent alignment (not using the alignment details in the original RGI SAM output), the reference file of all ARG sequences is also required (since these are not included in the SAM files).

Generating the 'variant/mutant' ARG list

This step is not part of the pipeline *per se*, but needs to be performed once to generate a simple look-up table where each ARG in the reference database has a boolean indicator of whether or not it belongs to a particular category (term ARO:0000031). In ARO, each ARG is identified by an ARO term as well as a name, but there are many other terms which represent parent classes or other categories.

The ARO is essentially hierarchical, with parent, grandparent etc terms' properties thus conferred on child terms (such as ARGs) via "subclass", "part_of" relationships etc. However, it is not strictly a tree because some terms (mainly ARGs themselves) can have multiple parents. To take an example in the current release, the ARG named "Klebsiella mutant PhoP conferring antibiotic resistance to colistin" (assigned the ARO term 3003585) has relationships in the ontology partly summarised as follows:

- great-grandchild of "protein(s) and two-component regulatory system modulating antibiotic efflux" (ARO:3000451)
- child of "gene altering cell wall charge" (ARO:3003580)
- child of "antibiotic resistant gene variant or mutant" (ARO:0000031)

The three terms above are all siblings, with the parent term "determinant of antibiotic resistance" (ARO:3000000). (For more details, refer to: https://www.ebi.ac.uk/ols/ontologies/aro/terms?iri=http%3A%2F%2Fpurl.obolibrary.org%2Fobo%2FARO_3003585&viewMode=All&siblings=false ; ARO:3003585 is shown four times due to another of its parents, PhoP ARO:3000834 having both subclass and part_of relationships.)

In contrast the ARG "pgpB" (ARO:3003920) is a descendant term of "gene altering cell wall charge" (ARO:3003580) but not of ARO:0000031 nor any other terms at that level (children of ARO:3000000).

The object is therefore to simply trace each ARG term's ancestry to determine whether this contains the ARO:0000031 term or not.

a) The first step was to generate a list of all of the ARO terms specific to genes which might occur in the RGI output. One source of these terms is the first column of the `aro_index.tsv` file supplied with the CARD. We noticed that a very few of the ARG terms in the RGI results did not have entries in this file (dated 2nd March 2020). We therefore supplemented this by obtaining the then-current ontology (OWL XML format file `aro.owl`, obtained from https://www.ebi.ac.uk/ols/ontologies/aro on 5th October 2020) , and processed these as follows to obtain the union of the two files (as the new version might lack a few terms deprecated since the CARD release):

```
grep '<!-- http://purl.obolibrary.org/obo/ARO' aro.owl | \
  cut -f 2 -d_ | cut -f 1 -d' ' | sort -u | \
    awk '{ print "ARO:" $1 }'   > tmp_aro_terms.dat


echo "ARO Accession" > all_aro_terms.dat


cut -f 1 aro_index.tsv | grep -v 'ARO Accession' \
    >> tmp_aro_terms.dat

sort -u tmp_aro_terms.dat >> all_aro_terms.dat
```

b) The second step was to automatically use each of the ARO terms in the above file as queries of an upward traversal of the ARO. The script `classify_arg_aro.pl` was written for this purpose and was used thus:

```
classify_arg_aro.pl --owl owl.xml \
    --ontology_root 1000001 --terminal 0000031 \
    --query_file all_aro_terms.dat -comment 'ARO Accession' \
    --sub_delimiter ':' -subfield 1 -verbosity 0 \
    --out_prefix 'ARO:' --missing ABSENT_FROM_ONTOLOGY \
    > clsfy_all_terms_0000031_or_not.tsv \
    2> clsfy_all_terms_0000031_or_not.stderr
```

A copy of clsfy_all_terms_0000031_or_not.tsv was then places in the `data/args_info/` directory. This is a two-column file of the form:

```
ARO:3003917     ARO:0000031
ARO:3003918     ARO:1000001
ARO:3003919     ARO:1000001
ARO:3003920     ARO:1000001
...
...
ARO:3003583     ARO:1000001
ARO:3003585     ARO:0000031
ARO:3003588     ARO:1000001
```

(etc)

where the second column contains either an ontology 'root' node term (ARO:1000001) or the ARO:0000031 term (also, in a very small number of cases, the ARG term could not be found, possibly because it originated from the earlier `aro_index.tsv` but had been deprecated prior to our obtaining `aro.owl`).

For illustrative purposes only, the earlier examples can be interrogated interactively using the same script to determine which term(s) at a particular level (child of ARO:3000000) those ARGs belong:

```
classify_arg_aro.pl --owl aro.owl --query_node 3003585 \
    --termini_parent 3000000 --out_prefix ARO:
```

(output:)

```
query IDs to search
#query_node     terminal/root_node(s)
ARO:3003585     ARO:0000031,ARO:3000451,ARO:3003580
```

```
classify_arg_aro.pl --owl aro.owl --query_node 3003920 \
    --termini_parent 3000000 --out_prefix ARO:
```

(output:)

```
1 query IDs to search
#query_node     terminal/root_node(s)
ARO:3003920     ARO:3003580
```

Use `classify_arg_aro.pl --man` for further details. Dependencies include Getopt::Euclid, List::Compare, OWL::Simple::Parser .

Applying the variant/mutant filter in the pipeline

The stages of the mutant/vatiant filter are implemented in the Nextflow scripts `scrn_wrt_sqs_rgi_bwt_pairs.nf` (which also performs the previous standard filter) -and `scrn_aro_pcid_rgi_bwt_pairs.nf` . The former runs an R script to generate sequence files from the reads data, then a Perl script to generate independent alignments (using EMBOSS `water`) of these sequences with their reference ARG sequences and also a table summarising the properties of these alignments (including sequence identities). The second Nextflow script uses these properties conditionally to perform a screen (via another R script) depending on the ARG type, using the look-up table described in the previous section. It also creates a tabular format file where each row represents one ARG, using the same R script used in the standard filter.

3) Read-pair count screen

The final filter was trivial in execution but with the important rationale that within any sample, each ARG must have been identified by at least two independent observations (read pairs which passed all of the earlier filters). The definition here is that both reads of the pair must not only have passed the filtering criteria but also have been mapped to the same ARG (by name; some ARG names are represented by multiple reference sequences). This is therefore a strict criterion as some true read matches may be discarded if their partner read matches an adjacent ARG in a genome.

The production per-ARG data from filter (2) are the *_scrn2_argstats.tsv files (see *Nextflow* under *Scripts*); the number of read-pairs is in the third column. Using the example of sample #6400, this would be:

```
awk '$3 > 1' data/filtered/6640_scrn2_argstats.tsv \
      > data/filtered/6640_scrn3_argstats.tsv
```

or is trivially performed in R etc.

(this particular example would produce a final per-ARG file of 0 rows, since `6640_scrn2_argstats.tsv` contains two rows only, in which the number of pairs is 1 in both cases). This was not implemented in a Nextflow pipeline script.

Pipeline

The execution of RGI itself and of the scripts which implement the subsequent filtering procedure were coordinated via several Nextflow scripts (Figure S10). Some of these scripts will benefit from improvements in efficiency, and potentially reworking into a single workflow script. However, in the interests of reproducibility, these are described as in the configuration used, as a record of how the data were analysed.

Overview

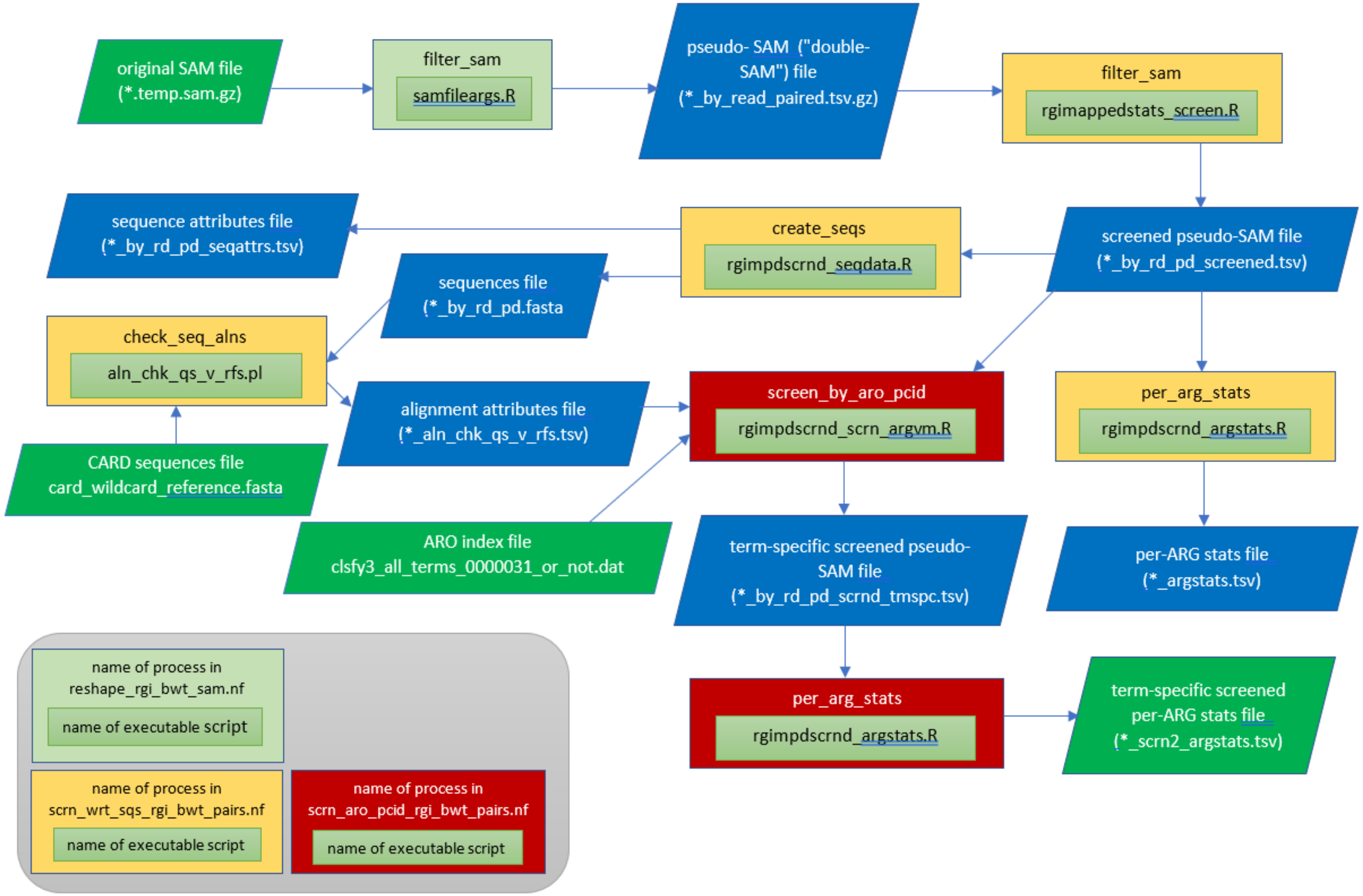

Figure S10. Pipeline for filtering RGI bwt SAM output files. Rectangles represent scripts (inner rectangles) and the Nextflow processes which run them (outer; colour-coded by Nextflow script). Parallelograms represent data files (input or production output termini in green). Wildcards '*' denote that there is one file per sample.

Scripts

Nextflow

These scripts were run using Nextflow version 20.04.1 build 5335.

Reference: di Tommaso *et al.* (2017) doi:10.1038/nbt.3820

`rgi_bwt_from_list.nf`

This script executes RGI as previously described (see section *Running RGI*). Processing time was 245 CPU hours on several 80 x 2.4 GHz or 144 x 2.5 GHz CPU core Linux servers.

This script uses a basic means of specifying which samples to process, as originally it was designed to be run independently on multiple servers; jobs are not despatched to servers via a batch queue. Given that the servers may be accessing the same directory on a shared disk, the script does not process all of the FASTQ paired files present, but only those whose names correspond to the sample IDs in a list file. So a list of all sample IDs was simply split into multiple subset lists of IDs. If using only 1 server, or > 1 server via a batch queue, the script may be better modified to a more orthodox approach where each pair of FASTQ files is picked up and processed as and when via the Nextflow platform.

Example execution:

```
nextflow run rgi_bwt_from_list_nforks.nf \
      --list_file data/samples_info/samples_file.00.lst \
      --n_threads 32 --storage_dir data/rgi_output
```

The remaining scripts implement the filtering (screening) procedure (see section *Filtering the RGI results by alignment properties*).

`reshape_rgi_bwt_sam.nf`

This script constitutes part of the 'standard filtering'.

This script also uses a basic means of specifying which samples to process, as it was run independently on multiple servers; refer to rgi_bwt_from_list.nf for details.

This runs the `samfileargs.R` script once for each sample. Processing time was 51 CPU hours on the same hardware previously described.

Example execution:

```
nextflow run reshape_rgi_bwt_sam.nf \
      --list_file data/samples_info/samples_file.00.lst
```

This would by default read input from `data/rgi_output` and generate output files (two tables per sample) in the default directory `data/rgi_sam_reshaped/` .

`scrn_wrt_sqs_rgi_bwt_pairs.nf`

("Screen, write sequences from the RGI bwt-originating paired-format files".)

This script implements the remainder of the 'standard filtering' stages (and also generates data files used subsequently by the 'variant/mutant filter'). It runs the following scripts:

- `rgimappedstats_screen.R`

- `rgimpdscrnd_seqdata.R`
- `aln_chk_qs_v_rfs.pl`
- `rgimpdscrnd_argstats.R`

The last of these is also run by the next workflow script.

Processing time was 20 CPU hours on the same hardware previously described.

Example execution:

```
nextflow run scrn_wrt_sqs_rgi_bwt_pairs.nf --maxforks 12
```

This would read input from `data/rgi_sam_reshaped/` by default and generate output files (two tables per sample) in the default directory `data/filtered/` .

`scrn_aro_pcid_rgi_bwt_pairs.nf`

("Screen conditionally on ARO-annotations by percentage identities in RGI bwt-originating paired-format files".)

This script implements the 'variant/mutant filtering' stage. It runs the following scripts:

- `rgimpdscrnd_scrn_argvm.R`
- `rgimpdscrnd_argstats.R`

Example execution:

```
nextflow run scrn_aro_pcid_rgi_bwt_pairs.nf --maxforks 12
```

By default this would read input from `data/filtered` and generate output files (two tables per sample) in the same directory. The final per-read-pair data are in the files *_by_rd_pd_scrnd_tmspc.tsv (by read, paired format, screened including (ARO) term-specific screen).

The final per-ARG data are in the files *_scrn2_argstats.tsv . In the latter files, the "read_pairs" column contains the numbers of read pairs where both R1 and R2 passed the filters *and were mapped to the same ARG name*; columns "R1_freq" and "R2_freq" refer to the forward and reverse reads individually (respectively columns 3, 10, 11). Values of each column can therefore be 0, depending on the values of the other columns.

R

These scripts were run using R version 4.0.1 (2020-06-06) "See Things Now".

These were written to be run automatically via a pipeline platform and are somewhat limited in flexibility regarding input and output filename formats.

`samfileargs.R`

This reshapes one an RGI bwt output SAM file into a new tabular format (two output tables), and calculates various alignment metrics which are also written to the new tables. The two output tables essentially contain the same data, but one consists of one line (record) per read; this is not used further. The other consists of one line per read-pair, and is used as input to the subsequent stage.

This script takes two arguments as input: the path of a SAM-format alignment file (output by RGI bwt; previous section) and the path of the directory in which output files will be created, in that order, e.g. using the previous example of sample ID '6400' (see *Running RGI*) and an output directory `data/rgi_sam_reshaped/` :

```
R --vanilla --slave --args \
      data/rgi_output/rgi_bwt_6400/rgi_bwt_6400.temp.sam.gz \
      data/rgi_sam_reshaped < scripts/R/samfileargs.R
```

The SAM file may be compressed (gzipped). The SAM files created by RGI bwt using BowTie2 contain one record (row) per read, with the forward (R1) and reverse (R2) reads in consecutive rows. SAM-format files created by other software may be used as long as they adhere to this; otherwise, the script will exit with an error condition. The RGI SAM files contain the reads which were not mapped to any reference (fields rname == "*" and pos == 0); a SAM file which omits these rows is also suitable as input. Segments that had been removed from reads had been soft-clipped by BowTie2 (not hard-clipped, which would lead to omission of the clipped nucleotides from the seq field, which would reduce accuracy of some of the calculated metrics and lead to over-lenient filtering in later steps). Development note: Use of SAM format was useful for development, but future improvements to efficiency may include parsing the BAM-format input instead using appropriate libraries.

The name of the SAM file should contain a string of digits, which is interpreted as the sample ID and determines the names of the output files.

The principal output file has a name of the form *`sampleID`*`_by_read_paired.tsv` . Each row represents one read pair, with most of the columns containing data specific to either the R1 or R2 read. A second output file is named *`sampleID`*`_by_read_unpaired.tsv`, in which each row represents one read, with pairs of reads in consecutive rows. This file is not used further here.

rgimappedstats_screen.R

This script performs the standard filter, and calculates some statistics from the output files. (It also creates some sequence-data files from the alignments, which are used later by the variant/mutant filter.) It takes two arguments as input: the path of the tabular file containing one read pair per row (output by samfileargs.R) and the path of the directory in which output files will be created, in that order, e.g. using the previous example of sample ID '6400' and the output directory `data/filtered/` :

```
R --vanilla --slave \
    --args data/rgi_sam_reshaped/6400_by_read_paired.tsv \
    data/filtered < scripts/R/rgimappedstats_screen.R \
    > 6400_by_rd_pd_screened.stdout
```

The name of the input file should begin with a string of digits, which is interpreted as the sample ID.

The principal output file has a name of the form *`sampleID`*`_by_rd_pd_screened.tsv` . The format is largely similar to that of the input file, but includes some additional columns,

including flags indicating which of the two reads (R1, R2) of a pair passed the filter. In every row, at least one of the two reads will have passed, with the double-failures having been discarded. At this stage, a read-pair is treated as passed if either or both of its reads have passed (irrespective of whether the reads were mapped to the same ARG).

The script is not optimally efficient for several reasons. In order to generate the production data for further analysis, the filters are applied serially, reducing the number of passed pairs at each stage (and could be applied in any order). However, with the aim of reporting summaries of the distributions of the various calculated parameters, in a separate procedure each filter is also applied independently to the initial input set of reads; this provides useful indications of the incidence of success or failure for each of the filters (this was useful for development purposes). Summaries are written to standard output.:

`rgimpdscrnd_argstats.R`

This is used in both the 'standard filter' (1) and also filter (2), the ARO-term specific 'variant/mutant' screen.

This takes three arguments as input: the path of the tabular file containing the standard-filtered read pairs, one pair per row (output by rgimappedstats.R) and secondly the path of the directory in which output files will be created. A third argument is the suffix of the output file name, which has a default value of '_argstats.tsv'. E.g. using the previous example of sample ID '6400' and the same output directory as the input director (`data/filtered/`):

```
R --vanilla --slave \
    --args data/filtered/6400_by_rd_pd_screened.tsv \
    data/filtered < scripts/R/rgimpdscrnd_argstats.R \
    > 6400_argstats.stdout
```

The name of the input file should begin with a string of digits, which is interpreted as the sample ID.

The principal output file has a name of the form *`sampleID`*`_argstats.tsv`, which contains one row per unique ARG name. The first three columns are the ARG name, the ARO term for the ARG, and the count of read pairs (where both R1 and R2 passed and mapped to this same ARG; this column can thus be zero). The remaining columns contain mean values for each of the metrics for all reads which passed and mapped to the ARG (for R1 and R2 individually). This file did not contribute to the final production incidence values, but was used for contrasting with the counterpart file generated from the additional term-specific screen (filter 2). Some summary statistics concerning read pairs which mapped to the same or different ARGs are written to standard output.

`rgimpdscrnd_seqdata.R`

The first stage of the 'vartiant/mutant' filter involves obtaining sequence files suitable as input for the following stage (independent alignment). This script takes two arguments as input: the path of the tabular file containing the standard-filtered read pairs, one pair per row (output by `rgimappedstats_screened.R`) and secondly the path of the directory in which output files will be created.

E.g. using the previous example of sample ID '6400' and the same output directory as the input directory ('data/filtered/'):

```
R --vanilla --slave \
    --args data/filtered/6400_by_rd_pd_screened.tsv \
    data/filtered < scripts/R/rgimpdscrnd_seqdata.R \
    > 6400_by_rd_pd_seqattrs.stdout
```

The principal output file has a name of the form *sampleID*_by_rd_pd.fasta, a FASTA-format file of the mapped sequence reads (whole sequences as input to RGI, not just the reference-matching segments). The sequence IDs are the same as the original read IDs but with colons replaced by underscores. The description field consists of a number of space-delimited fields, including the ID of the ARG reference sequence to which the read had been mapped. A second output file, with a name of the form *sampleID*_by_rd_pd_seqattrs.tsv, contains selected fields from the input tables, describing properties of each sequence read and how it was matched.

rgimpdscrnd_scrn_argvm.R

This takes 3 arguments to specify input: the tabular file containing the standard-filtered read pairs, one pair per row (output by rgimappedstats_screened.R); the table of pairwise sequence identities (*_aln_chk_qs_v_rfs.tsv, output by aln_chk_qs_v_rfs.pl); and the ARO annotations file(see *Generating the 'variant/mutant' ARG list*). A fourth argument specifies the output directory.

E.g. using the previous example of sample ID '6400' and the same output directory as the input directory ('data/filtered/'):

```
R --vanilla --slave --args 6640_by_rd_pd_screened \
     6640_aln_chk_qs_v_rfs.tsv \
     data/args_info/clsfy_all_terms_0000031_or_not.tsv \
     data/filtered < scripts/R/rgimpdscrnd_scrn_argvm.R
```

Perl

aln_chk_qs_v_rfs.pl

This requires the EMBOSS package to be installed. This is run via the required Perl packages BioPerl and BioPerl-Run (modules Bio::SeqIO, Bio::AlignIO, Bio::Factory::EMBOSS). (Also requires List::MoreUtils.) The purpose is to perform pairwise alignments of the reads and their corresponding, putatively-identified reference ARG sequences, independently of the alignment generated by the original identification. This is to calculate definitive sequence identities, for general assessment of predicted ARGs but also specifically for use by the conditional 'variant/mutant' screen.

This script takes two arguments as input: a FASTA file containing the reference ARG sequences, and the path of the FASTA file (*_by_rd_pd.fasta) containing the corresponding reference-sequence annotations (created by rgimpdscrnd_seqdata.R).

The reference sequences are assumed here to be in the file card_wildcard_reference.fasta, created during the loading of the

CARD/WildCARD database files into RGI in local mode; this file will then be in the directory `localDB/` relative to the working directory in which those rgi commands were run (see the section *Initialising RGI*).

The output data is in tabular format, written to standard output.

E.g. using the previous example of sample ID '6400' and the same output directory as the input directory ('`data/filtered/`'):

```
scripts/perl/aln_chk_qs_v_rfs.pl \
      database/localDB/card_wildcard_reference.fasta \
      data/filtered/6400_by_rd_pd.fasta \
      > data/filtered/6640_aln_chk_qs_v_rfs.tsv
```

Availability

The scripts are available at https://fred.fera.co.uk/jwalshaw/argisamfilter ("An RGI SAM filter").

Acknowlegment

We are grateful to McMaster University and the authors of RGI and CARD (Alcock *et al.*, 2020; https://doi.org/10.1093/nar/gkz935) for granting us a license to use their software and databases for this project.
